# Real-time estimation of high-resolution flow fields and reduced-order coordinates from event-based imaging velocimetry

**DOI:** 10.1007/s00348-026-04335-7

**Published:** 2026-10-29

**Authors:** Luca Franceschelli, Enrico Amico, Christian E. Willert, Marco Raiola, Gioacchino Cafiero, Stefano Discetti

**Affiliations:** 1 Department of Aerospace Engineering, Universidad Carlos III de Madrid, Avda. Universidad 30, 28911 Leganés, Madrid Spain; 2 Department of Mechanical and Aerospace Engineering, Politecnico di Torino, Corso Duca degli Abruzzi 24, 10129 Turin, Italy; 3 DLR Institute of Propulsion Technology, German Aerospace Center, Linder Höhe, 51170 Köln, Germany

## Abstract

A data-driven framework is proposed to estimate high-resolution (HR) velocity fields and reduced-order flow coordinates from coarse real-time event-based imaging velocimetry (rt-EBIV) measurements. Low-resolution (LR) data are retrieved through fast event analysis on a coarse grid. During offline training, the LR/HR mapping and a linear dynamical model in a proper orthogonal decomposition (POD)-based reduced-order space are identified. Online, each incoming LR snapshot is projected onto the LR basis, and the corresponding HR reduced-order coordinates are estimated and temporally regularized. Three estimators are proposed and compared: a direct Kalman filter (KF), a linear stochastic estimation (LSE) combined with Kalman filtering, and a variance-rescaled implementation (LSE+VR). The full HR velocity field is then reconstructed from the estimated reduced-order coordinates using the retained POD basis. The framework is assessed on two turbulent flows acquired by pulsed event-based imaging velocimetry (EBIV): a submerged water jet and a channel flow over a square rib. All three estimators outperform direct cubic interpolation of the LR fields, recovering physically consistent HR flow states with improved estimation of turbulent kinetic energy, spectral content, reduced-order dynamics, and temporal coherence. The linear stochastic estimator yields the lowest overall reconstruction error, while its variance-rescaled implementation provides nearly identical values and improves the recovery of fluctuation energy and higher-order content. The direct KF formulation remains highly efficient in terms of computational cost and provides the closest agreement with the HR reference in the temporal and spatial spectral analyses. The bulk of the computational cost lies in the full-field HR reconstruction, whereas the reduced-order coordinate estimation alone is negligible compared with the cost of the LR processing itself. The framework enables deliberately coarse real-time EBIV processing to be combined with reduced-order refinement, extending the effective real-time operating range towards higher update frequencies while still providing richer and dynamically more consistent HR flow representations. This makes the approach attractive for high-quality real-time flow diagnostics and as a first step towards future observer-based and closed-loop flow-control applications.

## Introduction

Particle image velocimetry (PIV) is widely used for experimental flow diagnostics because it provides non-intrusive, spatially resolved velocity measurements over an extended field of view (Raffel et al. 2018). Compared with pointwise sensing, full-field velocimetry provides direct access to the spatial distribution of the velocity field and to quantities that depend on it, such as gradients, shear, vorticity, and multi-point correlations. This information is central to physical interpretation and particularly valuable for reduced-order modelling and observer design, since it enables state descriptions that are linked to the spatial structure of the flow rather than inferred from a small number of local probes (Glauser and George 1987; Rajaee et al. 1994; Berkooz et al. 2003). For flow-control applications, spatially distributed velocity information is particularly attractive because it supports state representations that are more directly connected to the underlying dynamics than those inferred from a limited number of local measurements (Siegel et al. 2003; Willert et al. 2010; Gautier et al. 2015; Varon et al. 2019; Mccormick et al. 2024; Nonomura et al. 2025).

While PIV provides detailed spatially resolved information, it comes with limited temporal bandwidth and higher computational latency. In contrast, conventional probes (such as hot wires, pressure transducers, or microphones) are more suitable for real-time applications due to their high sampling rates, straightforward hardware integration, and minimal processing requirements. However, they provide only sparse spatial information and are often intrusive. Flow-control experiments have traditionally relied on point sensors (see e.g. Audiffred et al., 2024; Dacome et al., 2024), although their effective use generally requires careful sensor placement informed by prior knowledge of the flow, even in canonical configurations. Real-time imaging velocimetry could bridge this gap by combining the spatial information of velocity field measurements with the responsiveness needed for monitoring, estimation, and, ultimately, control.^1^

The attractiveness of implementing field information in flow-control strategies has motivated several studies aimed at advancing PIV towards real-time operation, including accelerated image-processing pipelines (Kreizer et al. 2010; Willert et al. 2010), optical-flow-based velocimetry (Gautier et al. 2015; Pimienta and Aider 2025; Bollt et al. 2025), and sparse or reduced-order reconstruction strategies (Kanda et al. 2022). However, real-time operation is generally obtained at the cost of either specialized hardware and tailored implementations, such as field-programmable gate array (FPGA)-based processing (Kreizer et al. 2010) or Graphics Processing Units (GPU) acceleration (Pimienta and Aider 2025), or a simplified measurement and processing chain. In the latter case, latency is reduced by accepting compromises such as coarser spatial resolution, fewer iterations, or lighter validation. Kanda et al. (2022) proposed sparse processing PIV (SPPIV) as a framework for real-time flow field estimation. Rather than processing the full image, cross-correlation is performed only at a small number of interrogation windows placed at optimally selected spatial locations. A pre-trained Kalman filter (KF), formulated in the proper orthogonal decomposition (POD) coefficient space, maps the resulting sparse velocity vectors onto a low-dimensional flow representation, from which the full velocity field is reconstructed. The method estimated a small number of modes owing to the limited number of simultaneous sparse measurements. The effectiveness of this approach for closed-loop flow control has been subsequently demonstrated by Nonomura et al. (2025) and Viguera et al. (2026), who employed the SPPIV-estimated velocity field as a real-time feedback signal at update frequencies of up to 2 kHz for plasma-actuator-based control. Their studies, respectively, achieved regulation of the velocity field towards prescribed targets and mitigation of flow separation over an airfoil.

Event-based vision (EBV) provides a new route to perform cost-effective flow field measurements in the kHz range. EBV replaces conventional frame-based acquisition with asynchronous brightness-change detection at the pixel level (Mahowald and Mead 1991; Lichtsteiner et al. 2008). An event sensor outputs a stream of sparse spatiotemporal events only when the local intensity change crosses a contrast threshold. Each event carries a polarity flag: positive events are triggered by a local brightness increase exceeding the contrast threshold, whereas negative events correspond to a brightness decrease. A comprehensive review of this sensing paradigm and applications is provided by Gallego et al. (2022). Compared with frame-based cameras, event sensors offer major advantages in terms of temporal responsiveness, low redundancy, reduced data transfer, and high dynamic range, making them attractive for flow diagnostics with real-time capability.

The first application of an EBV sensor to velocimetry was reported by Drazen et al. (2011), who demonstrated particle tracking in a 5-cm-diameter perspex pipe. Over the following decade, event cameras found use across a broad range of flow measurement scenarios, including Background Oriented Schlieren Method (BOS) (Shiba et al. 2024), microfluidics (Howell et al. 2020), and cardiac flows (Amico et al. 2026). Rusch (2023) and Rusch and Rösgen (2023) developed a real-time system for three-dimensional particle tracking at relatively high velocities, albeit with a hard upper bound on the number of tracks that can be reliably reconstructed. More recently, Willert (2025) proposed a dual-sensor approach that yields dense and accurate velocity fields even in high-speed flows, with demonstrated measurements in a free jet with exit velocities up to 180 m/s at laser pulse separations down to 100 ns.

Franceschelli et al. (2025) have shown that event-based imaging velocimetry (EBIV) measurements can support reduced-order flow estimation in turbulent configurations. Willert et al. (2026) demonstrated the use of EBIV for real-time jet-flow control, reaching real-time full-frame measurements at an average rate of 270 Hz with a single-pass cross-correlation analysis on pseudo-frames, thus partially sacrificing spatial resolution. This opens the following question: can coarse, data-efficient EBIV measurements be used to estimate, in real time, high-resolution velocity fields and their reduced-order coordinates, without requiring high-speed frame-based cameras, high-power laser illumination, or the computational cost of a full offline-grade processing pipeline?

The present work addresses this question through a reduced-order cross-resolution estimation framework. A key point is that the low-resolution (LR) and high-resolution (HR) fields do not come from different instruments or different acquisition systems. Both datasets are extracted from the same raw EBV event stream processed according to two different strategies: a lightweight single-pass procedure representative of real-time operation provides the LR input, whereas a more accurate offline multi-frame, multi-pass procedure provides the HR reference used for training and assessment. No conventional frame-based camera data are used at any stage.

The proposed real-time estimation methodology combines this paired LR/HR processing with POD-based reduced-order modelling. In the online estimation phase, each incoming LR snapshot is projected onto the full-rank LR basis and used to estimate the corresponding reduced-order HR state. Three estimators are considered to map the LR state into the HR state: a direct Kalman Filter (KF) formulation, a Linear Stochastic Estimation (LSE)-based estimator followed by Kalman filtering, and a variance-rescaled LSE variant (LSE+VR) designed to mitigate the attenuation of poorly observable modes. The reconstructed fields are compared against the reduced-order HR reference and against direct cubic interpolation of the LR field onto the HR grid, adopted as a purely spatial baseline.

In this sense, the LR measurements can be regarded as spatially distributed, non-intrusive probes that can be combined with data-driven methods to reconstruct the corresponding HR flow field (Tu et al. 2013; Discetti et al. 2019).

The framework is evaluated on two broadband turbulent flows: a submerged water jet at $Re_D = U_j D/\nu \approx 6,000$, and a channel flow over a square rib at $Re_H = U_{ch} H/\nu \approx 1,500$. Here, $U_j$ is the jet bulk velocity, $U_{ch}$ is the asymptotic channel-flow velocity, *D* is the nozzle exit diameter, *H* is the rib height, and ν is the kinematic viscosity. These two cases were selected because they exhibit energetically distributed, multi-scale dynamics and therefore provide a demanding testbed for cross-resolution estimation. In addition to instantaneous fields, the analysis includes mean and second-order statistics, temporal and spatial spectra, reduced-order modal dynamics, temporal autocorrelation, overall reconstruction error, and computational cost.

The complete measurement and estimation workflow presented in this work is released as *VIBEstream*, an open-source Python package providing a simple interface for event-camera recording, live streaming, quick PIV-style processing, and real-time EBIV with direct velocity field output (see the code availability statement).

The remainder of the paper is organized as follows. Section 2 introduces the common reduced-order formulation and the three estimation methods. Section 3 describes the experimental datasets and the EBIV processing chain used to generate the paired LR and HR fields. Section 4 presents the reconstruction results and the computational cost analysis. Finally, Sect. 5 summarizes the work and discusses the practical implications, limitations, and possible extensions of the proposed framework.

## Methodology

**Fig. 1:**
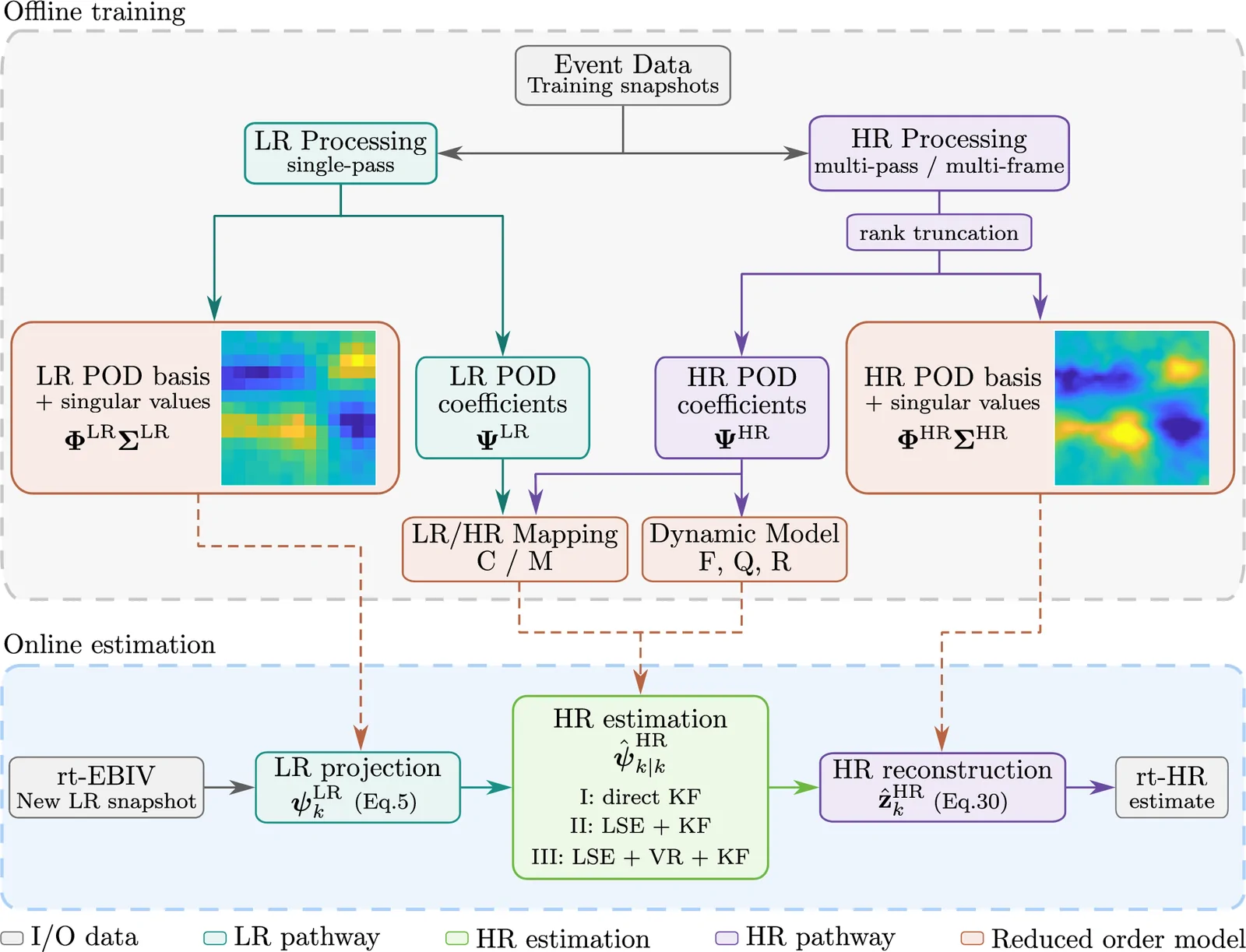
Offline training (top, grey dashed frame) and online estimation (bottom, blue dashed frame) pipeline. The colour coding distinguishes input/output data (grey), the LR pathway (teal), the HR pathway (purple), the reduced-order model operators (orange), and the HR estimation stage (green); dashed orange arrows denote the operators identified offline and deployed online. The abbreviation “rt” stands for real-time

This section describes the three estimation methodologies considered in the present work. Although they differ in the way the HR coefficients are estimated and temporally regularized, all three methods share the same offline/online framework and use the temporal coefficients from LR measurements as input.

For clarity, the main terminology used throughout the paper is defined here. The *latent coordinates* are the POD temporal coefficients, i.e. the modal amplitudes. The *reduced-order coordinates* denote the subset of these coefficients retained after truncation of the POD basis. Accordingly, the *latent space*, or equivalently the *reduced-order space*, is the subspace spanned by the retained POD modes. In this work, *state estimation* refers to the estimation of the retained POD coefficients from coarse real-time measurements. Finally, the *modal energy* denotes the POD modal energy, defined as the squared singular value associated with each mode.

The workflow pipeline is reported in Fig. 1. The main structure is divided into two phases:

- **Offline training**: event sequences are analysed both with a simple coarse analysis based on single-pass cross-correlation with non-overlapping sampling window (LR dataset) and with an advanced multi-pass multi-frame process (HR dataset). A mapping is then established between the LR and HR data with the aid of reduced-order coordinates.
- **Online estimation** : the stream of events is processed with the same quick evaluation process used to obtain the LR dataset. The data are then used to estimate the HR velocity fields using the mapping learned during training.

More explicitly, in the offline phase (top, grey dashed frame of Fig. 1) the recorded event data are processed by both pipelines: the single-pass LR processing and the multi-pass, multi-frame HR processing, the latter followed by the rank truncation discussed in Sect. 2.1. POD applied to the two datasets yields the LR basis and singular values $\boldsymbol{\Phi }^{\textrm{LR}}\boldsymbol{\Sigma }^{\textrm{LR}}$ with the associated coefficients $\boldsymbol{\Psi }^{\textrm{LR}}$ and their truncated HR counterparts. From the paired coefficient histories, the LR/HR mapping operator and the dynamics model matrices are identified. In the online phase (bottom, blue dashed frame), each new LR snapshot delivered by the real-time EBIV processing is projected onto the LR basis, Eq. (5); the reduced-order HR state $\hat{\boldsymbol{\psi }}^{\textrm{HR}}_{k|k}$ is then estimated through one of the three methods of the following sections, using the operators learned offline (dashed orange arrows in the figure); finally, when the full field is required, the HR snapshot is reconstructed through the retained HR basis.

From a practical standpoint, once the training is completed, the online stage operates as a self-contained estimator: each incoming LR snapshot is converted, within the available time budget, into an estimate of the reduced-order HR coordinates and, if required, into the corresponding full HR field. In a prospective observer-based or closed-loop flow-control architecture, the estimated reduced-order coordinates would play the role of the plant state: they could be fed directly to a state-feedback controller or serve as the measurement input of a higher-level observer, without requiring the full-field reconstruction in the loop.

A key point of the present framework is that the LR and HR velocity fields do not originate from different measurement systems. In both datasets, the same raw EBV event stream is processed through two distinct velocimetry pipelines of different computational cost and accuracy. The first is a lightweight single-pass procedure designed to emulate a realistic real-time implementation and provides the LR input to the estimator. The second is a more accurate offline multi-frame, multi-pass procedure and provides the HR reference fields used for training, validation, and performance assessment. No conventional frame-based camera data are used at any stage of the present work.

The main hypothesis is that a LR-HR mapping can be established by using a compact mapping in latent coordinates. This approach is in line with previous works exploiting reduced-order representations and/or nonlinear mappings for super-resolution (Deng et al. 2019; Fukami et al. 2021; Cortina-Fernández et al. 2021; Tirelli et al. 2023).

In this section, the common formulation is introduced first, followed by the specific description of each method. First, the POD implementation is briefly summarized, as it is used to identify the temporal coordinates for the mapping between LR and HR measurements, and for the state transitions. Later, the implementation of state observers for HR full-state estimation is presented.

### Proper orthogonal decomposition and shared framework

Following the snapshot method introduced by Sirovich (1987), POD is applied to the HR training dataset in fluctuation form. We denote the instantaneous velocity field as

1 $$\begin{aligned} \begin{aligned}&\textbf{U}(\textbf{x},t)= \overline{\textbf{U}}(\textbf{x})+\textbf{u}(\textbf{x},t)\,, \end{aligned} \end{aligned}$$

where $\overline{\textbf{U}}(\textbf{x})=\langle \textbf{U}(\textbf{x},t)\rangle _T$ denotes the temporal mean field and $\textbf{u}(\textbf{x},t)$ the corresponding fluctuation field according to the Reynolds decomposition. In PIV measurements, each snapshot samples the *c* components of the fluctuating velocity field over a grid of sampling points covering the measurement domain. Let $N_p^{\textrm{HR}}$ and $N_p^{\textrm{LR}}$ denote the number of spatial sampling points of the HR and LR velocity fields, respectively. Then, $\textbf{z}^{\textrm{HR}}_k \in \mathbb {R}^{c\,N_p^{\textrm{HR}}}$ and $\textbf{z}^{\textrm{LR}}_k \in \mathbb {R}^{c\,N_p^{\textrm{LR}}}$ denote the corresponding *k*-th HR and LR fluctuation snapshots. Each snapshot is formed by stacking all the velocity components over all the available sampling points into a single column vector. Denoting by $\textbf{Z}^{\textrm{HR}} \in \mathbb {R}^{c N_p^{\textrm{HR}} \times N_t}$ the matrix collecting the $N_t$ HR training snapshots, its economy-sized singular value decomposition is

2 $$\begin{aligned} \textbf{Z}^{\textrm{HR}} = \left[ \textbf{z}^{\textrm{HR}}_1,\ldots ,\textbf{z}^{\textrm{HR}}_{N_t} \right] = \mathbf {\Phi }^{\textrm{HR}} \mathbf {\Sigma }^{\textrm{HR}} \mathbf {\Psi }^{\textrm{HR}^{\scriptstyle \textsf{T}}}, \end{aligned}$$

where $\mathbf {\Phi }^{\textrm{HR}} \in \mathbb {R}^{c N_p^{\textrm{HR}} \times m}$ contains the spatial modes, $\mathbf {\Psi }^{\textrm{HR}} \in \mathbb {R}^{N_t \times m}$ the temporal modes, $\mathbf {\Sigma }^{\textrm{HR}} \in \mathbb {R}^{m \times m}$ the singular values, and $m \le \min (N_t, c N_p^{\textrm{HR}})$ the maximum rank of $\textbf{Z}^{\textrm{HR}}$. A reduced-order representation of the HR field can be obtained through a Low-Order Reconstruction (LOR) by truncating the HR latent space from rank *m* to rank *r*, retaining only the first *r* modes:

3 $$\begin{aligned} \textbf{Z}^{\textrm{HR}} \approx \boldsymbol{\Phi }^{\textrm{HR}}_r \boldsymbol{\Sigma }^{\textrm{HR}}_r \boldsymbol{\Psi }^{\textrm{HR}^{\scriptstyle \textsf{T}}}_r\,, \end{aligned}$$

where $\boldsymbol{\Phi }^{\textrm{HR}}_r \in \mathbb {R}^{c N_p^{\textrm{HR}} \times r}$ contains the retained spatial modes, $\boldsymbol{\Sigma }^{\textrm{HR}}_r \in \mathbb {R}^{r \times r}$ is the corresponding diagonal matrix of singular values, and $\boldsymbol{\Psi }^{\textrm{HR}}_r \in \mathbb {R}^{N_t \times r}$ contains the associated temporal modes. Hereafter, $\boldsymbol{\psi }^{\textrm{HR}}_k \in \mathbb {R}^{r}$ denotes the reduced HR coefficient vector associated with the *k*-th snapshot, i.e. the *k*-th row of $\boldsymbol{\Psi }^{\textrm{HR}}_r$ transposed to a column vector.

The choice of the truncation rank involves a delicate trade-off between preserving physically meaningful flow structures and rejecting noise, which typically contaminates the higher-order modes. In this sense, previous studies have proposed criteria based on the modal amplitude relative to the measurement noise (Epps and Techet 2010), on the degree of spatial organization retained by the modes (Brindise and Vlachos 2017), and on the progressive loss of modal reliability as noise corruption increases (Epps and Krivitzky 2019). In this work, the truncation rank *r* is selected using the *elbow criterion*, restated by Raiola et al. (2015) as the relative decrease rate of the reconstruction error at the *i*-th POD mode, *F*(*i*), rather than on a prescribed retained energy threshold. The threshold F(i)=0.999 is adopted to determine the number of retained HR modes. This threshold identifies the mode index beyond which adding one more mode reduces the reconstruction error by less than 0.1% in relative terms, and the additional modes are expected to be dominated by measurement noise. Other values were also considered: stricter truncations discard part of the energetic broadband content of the present flows, whereas milder ones mostly add noise-dominated modes with no appreciable benefit on the estimation. The relatively high retained ranks (r≈180–190) reflect the broadband, multi-scale character of the two datasets, whose slowly decaying modal energy distribution (see Sect. 4) requires many modes to represent the resolved dynamics, rather than the specific threshold choice. Note also that the POD truncation acts at the same time as an effective denoising stage, since measurement noise is mostly scattered across the discarded low-energy modes (Raiola et al. 2015; Brindise and Vlachos 2017).

An analogous POD analysis is applied to the LR training dataset. Denoting by $\textbf{Z}^{\textrm{LR}} \in \mathbb {R}^{cN_p^{\textrm{LR}} \times N_t}$ the matrix of rank *n* collecting the LR training snapshots, its singular value decomposition yields

4 $$\begin{aligned} \textbf{Z}^{\textrm{LR}} = \boldsymbol{\Phi }^{\textrm{LR}} \boldsymbol{\Sigma }^{\textrm{LR}} \boldsymbol{\Psi }^{\textrm{LR}^{\scriptstyle \textsf{T}}}\,. \end{aligned}$$

In contrast to the HR side, no truncation is applied to the LR latent space, and the full numerical rank *n* is retained.

During the validation and online reconstruction stage, i.e. for the snapshots belonging to the testing subset, each incoming LR snapshot $\textbf{z}^{\textrm{LR}}_k$ is projected onto the previously identified full-rank LR basis to obtain the coefficient vector

5 $$\begin{aligned} \boldsymbol{\psi }^{\textrm{LR}}_k = \boldsymbol{\Sigma }^{\textrm{LR}^{\scriptstyle -1}} \boldsymbol{\Phi }^{\textrm{LR}^{\scriptstyle \textsf{T}}} \textbf{z}^{\textrm{LR}}_k \in \mathbb {R}^{n}{,} \end{aligned}$$

which constitutes the common input to all three estimation methodologies described below.

In the following, we consider two-component velocity measurements, i.e. $c=\text {2}$, although the extension to volumetric three-component data is straightforward.

### Estimation of HR states from LR measurements

In this section, three estimators will be described:

- **Direct Kalman filter estimation**: the HR temporal coefficients are obtained using directly the LR data as observables for the Kalman filter.
- **LSE-based estimation with Kalman filtering**: the HR coefficients are first estimated with an LSE from the LR coefficients, and then a Kalman filter is applied as a regularizer.
- **Variance-rescaled LSE-based estimation**: based on the same strategy of the previous method, corrected with a mode-variance correction before the Kalman filtering to compensate for systematic attenuation of the least observable HR modes.

The three estimators differ in the way the reduced HR coefficients are inferred from the LR measurements. However, all of them rely on the same linear reduced-order state-transition model of the HR coefficients. This common dynamical model is introduced first, and the three estimation strategies are described subsequently.

For the estimator identification, it is convenient to collect the reduced coordinates at all time instants as column-wise data matrices,

6 $$\begin{aligned} \begin{aligned} \textbf{A}^{\textrm{HR}}&\equiv \left[ \boldsymbol{\psi }^{\textrm{HR}}_1,\ldots , \boldsymbol{\psi }^{\textrm{HR}}_{N_t} \right] = \boldsymbol{\Psi }^{\textrm{HR}\textsf{T}}_r \in \mathbb {R}^{r \times N_t}, \\ \textbf{A}^{\textrm{LR}}&\equiv \left[ \boldsymbol{\psi }^{\textrm{LR}}_1,\ldots , \boldsymbol{\psi }^{\textrm{LR}}_{N_t} \right] = \boldsymbol{\Psi }^{\textrm{LR}\textsf{T}} \in \mathbb {R}^{n \times N_t}. \end{aligned} \end{aligned}$$

These matrices are introduced only as a compact notation for the state estimation stage: each column corresponds to one time sample of the reduced HR state or LR measurement.

#### Common state-transition model

During online estimation, a state-transition model is used to regularize the output. Considering the short prediction horizon (only one step ahead in time-resolved measurements), a linear discrete one-step model is adopted for the reduced HR state. Specifically, the retained HR temporal coefficients are assumed to evolve according to a linear state-transition matrix $\textbf{F} \in \mathbb {R}^{r \times r}$, such that

7 $$\begin{aligned} \boldsymbol{\psi }^{\textrm{HR}}_{k+1} \approx \textbf{F}\,\boldsymbol{\psi }^{\textrm{HR}}_k. \end{aligned}$$

To estimate $\textbf{F}$, two matrices collecting consecutive reduced-order snapshots are defined as

8 $$\begin{aligned} \begin{aligned} \textbf{A}^{\textrm{HR},-}&= \left[ \boldsymbol{\psi }^{\textrm{HR}}_1 \;\cdots \; \boldsymbol{\psi }^{\textrm{HR}}_{N_t-1} \right] \in \mathbb {R}^{r \times (N_t - 1)}\,,\\ \textbf{A}^{\textrm{HR},+}&= \left[ \boldsymbol{\psi }^{\textrm{HR}}_2 \;\cdots \; \boldsymbol{\psi }^{\textrm{HR}}_{N_t} \right] \in \mathbb {R}^{r \times (N_t - 1)}, \end{aligned} \end{aligned}$$

The matrix $\textbf{F}$ is then obtained in the least-squares sense as

9 $$\begin{aligned} \textbf{F}= \textbf{A}^{\textrm{HR},+} \left( \textbf{A}^{\textrm{HR},-} \right) ^{\dagger }\,, \end{aligned}$$

where $(\cdot )^{\dagger }$ denotes the Moore–Penrose pseudoinverse. Therefore, $\textbf{F}$ represents the best-fit linear evolution operator of the reduced HR state over one time step.

#### Method I—Direct Kalman filter estimation

The first estimator directly infers the reduced HR state from the LR coefficient vector through a linear state-space model formulated entirely in coefficient space. Since the estimation is directly embedded in the Kalman filtering process, this method will be referred to as KF.

The reduced HR state is assumed to evolve according to

10 $$\begin{aligned} \boldsymbol{\psi }^{\textrm{HR}}_k = \textbf{F}\,\boldsymbol{\psi }^{\textrm{HR}}_{k-1} + \textbf{w}_k\,, \end{aligned}$$

where $\textbf{w}_k$ denotes the process noise of the Kalman filter state model, accounting for unmodelled dynamics and mismatch between the true reduced-order evolution and its one-step linear approximation through $\textbf{F}$. It is assumed to be zero mean with covariance $\textbf{Q}$, estimated offline from the residuals of the identified linear model. The measurement model relates the LR coefficients vector to the reduced HR state as

11 $$\begin{aligned} \boldsymbol{\psi }^{\textrm{LR}}_k = \textbf{C}\,\boldsymbol{\psi }^{\textrm{HR}}_k + \boldsymbol{\eta }_k\,, \end{aligned}$$

where $\textbf{C} \in \mathbb {R}^{n \times r}$ is a linear measurement operator mapping HR coefficients to LR coefficients, and $\boldsymbol{\eta }_k$ denotes the measurement noise, assumed zero mean with covariance $\textbf{R}$.

The comparison between the HR prediction and the LR measurement is deliberately performed in the LR coefficient space. Indeed, mapping a LR measurement back to the HR space before comparison would amount to solving an inverse problem, which is intrinsically ill-posed because the LR data do not contain the missing high-frequency content. By contrast, projecting the predicted HR state into the LR space through $\textbf{C}$ is a well-posed forward operation. The resulting mismatch is then mapped back to the HR state through the covariance-weighted Kalman gain, ensuring a stable and consistent correction step.

The operator $\textbf{C}$ is identified offline from the training data by ridge-regularized least squares,

12 $$\begin{aligned} \textbf{C} = \textbf{A}^{\textrm{LR}}_{\textrm{train}} \textbf{A}^{\textrm{HR}^{\scriptstyle \textsf{T}}}_{\textrm{train}} \left( \textbf{A}^{\textrm{HR}}_{\textrm{train}} \textbf{A}^{\textrm{HR}^{\scriptstyle \textsf{T}}}_{\textrm{train}} + \lambda \textbf{I}_r \right) ^{-1}\,, \end{aligned}$$

where λ is a small regularization parameter, here set to $\lambda = 10^{-12}$.

The process noise covariance $\textbf{Q}$ is estimated empirically from the residuals of the one-step linear model on the training set. Specifically, the matrix

13 $$\begin{aligned} \textbf{E}_{\textrm{model}} = \textbf{A}^{\textrm{HR},+} - \textbf{F}\,\textbf{A}^{\textrm{HR},-} \end{aligned}$$

collects, column by column, the sample residuals of the reduced-order state equation, i.e. the observed realizations of the process noise term $\textbf{w}_k$ over the training set. The corresponding covariance estimate is then

14 $$\begin{aligned} \textbf{Q} = \frac{1}{N_t - 1} \textbf{E}_{\textrm{model}}\textbf{E}_{\textrm{model}}^\textsf{T}\,. \end{aligned}$$

Similarly, the measurement noise covariance $\textbf{R}$ is estimated from the residuals of Eq. (11) over the validation subset. Denoting by $\textbf{A}^{\textrm{LR}}_{\textrm{val}}$ and $\textbf{A}^{\textrm{HR}}_{\textrm{val}}$ the matrices collecting the corresponding LR and HR coefficients vectors on that subset, one defines

15 $$\begin{aligned} \textbf{E}_{\textrm{meas}} = \textbf{A}^{\textrm{LR}}_{\textrm{val}} - \textbf{C}\,\textbf{A}^{\textrm{HR}}_{\textrm{val}}\,. \end{aligned}$$

The matrix $\textbf{E}_{\textrm{meas}}$ therefore collects, column by column, the sample residuals of the measurement equation and is used as an empirical collection of realizations of the measurement noise term $\boldsymbol{\eta }_k$. The corresponding covariance estimate is

16 $$\begin{aligned} \textbf{R} = \frac{1}{N_{\textrm{val}} - 1} \textbf{E}_{\textrm{meas}}\textbf{E}_{\textrm{meas}}^\textsf{T}\,. \end{aligned}$$

Once $\textbf{F}$, $\textbf{C}$, $\textbf{Q}$, and $\textbf{R}$ have been identified, the reduced HR state is estimated online through the classical Kalman prediction-update procedure. At each k-th timestep, the Kalman gain is updated as

17 $$\begin{aligned} \textbf{K}_k = \textbf{P}_{k|k-1}\,\textbf{C}^\textsf{T} \left( \textbf{C}\,\textbf{P}_{k|k-1}\,\textbf{C}^\textsf{T} + \textbf{R} \right) ^{-1}\,, \end{aligned}$$

where $\textbf{P}_{k|k-1}$ denotes the covariance matrix of the *a priori* estimation error associated with the predicted reduced HR state. Although, in principle, this quantity corresponds to the covariance of the difference between the true state and its prediction, the true state $\boldsymbol{\psi }^{\textrm{HR}}_k$ is unknown during online estimation. Therefore, $\textbf{P}_{k|k-1}$ is computed recursively by propagating the previous updated covariance through the state-transition model and adding the process noise covariance, namely

18 $$\begin{aligned} \textbf{P}_{k|k-1} =\textbf{F}\,\textbf{P}_{k-1|k-1}\,\textbf{F}^\textsf{T} + \textbf{Q}\,. \end{aligned}$$

The corrected estimate can then be written compactly as

19 $$\begin{aligned} \begin{aligned} \hat{\boldsymbol{\psi }}^{\textrm{HR}}_{k|k}&= \textbf{F}\,\hat{\boldsymbol{\psi }}^{\textrm{HR}}_{k-1|k-1} + \textbf{K}_k \left( \boldsymbol{\psi }^{\textrm{LR}}_k - \textbf{C}\,\textbf{F}\,\hat{\boldsymbol{\psi }}^{\textrm{HR}}_{k-1|k-1} \right) \,, \end{aligned} \end{aligned}$$

which highlights that the updated HR state is obtained by correcting the model-based prediction through the mismatch between the measured LR coefficients and the corresponding projection of the predicted HR state from the model. The associated covariance matrices are updated according to the standard Kalman filter recursion (Kalman 1960; Welch and Bishop 1995). In the present implementation, the initial state is obtained by applying the pseudoinverse of $\textbf{C}$ to the first available LR measurement.

#### Method II—LSE-based estimation with Kalman filtering

The second approach decouples the spatial mapping from the temporal regularization. A LSE is first used to map the LR coefficient vector directly onto an instantaneous estimate of the reduced HR state. A Kalman filter is then applied only as a temporal denoising and regularization stage. This method is hereinafter referred to as *LSE*.

The LSE operator $\textbf{M} \in \mathbb {R}^{r \times n}$ is identified offline from the training data by ridge-regularized least squares,

20 $$\begin{aligned} \textbf{M} = \textbf{A}^{\textrm{HR}}_{\textrm{train}} \textbf{A}^{\textrm{LR}^{\scriptstyle \textsf{T}}}_{\textrm{train}} \left( \textbf{A}^{\textrm{LR}}_{\textrm{train}} \textbf{A}^{\textrm{LR}^{\scriptstyle \textsf{T}}}_{\textrm{train}} + \lambda \textbf{I}_{n} \right) ^{-1} \end{aligned}$$

where λ is the same regularization parameter introduced in Eq. (12) and set also in this case to $\lambda = 10^{-12}$. In contrast to $\textbf{C}$ in Eq. (12), which is used in Method I as a forward operator to predict the LR coefficients associated with a given reduced HR state, $\textbf{M}$ directly provides a statistical LR-to-HR mapping and is therefore used here to reconstruct an instantaneous estimate of the reduced HR coefficients from the observed LR ones.

At each time step, the instantaneous LSE estimate of the reduced HR coefficients is obtained as

21 $$\begin{aligned} \tilde{\boldsymbol{\psi }}^{\textrm{HR}}_k = \textbf{M}\,\boldsymbol{\psi }^{\textrm{LR}}_k\,. \end{aligned}$$

This estimate is optimal in the regularized least-squares sense over the training data, but it remains affected by frame-to-frame noise inherited from the LR measurement and tends to attenuate the variance of the least observable higher-order modes.

The Kalman filter is then introduced only as a temporal filtering stage acting on the LSE-based estimate. The reduced HR state is assumed to evolve according to

22 $$\begin{aligned} \boldsymbol{\psi }^{\textrm{HR}}_k = \textbf{F}\,\boldsymbol{\psi }^{\textrm{HR}}_{k-1} + \textbf{w}_k\,, \end{aligned}$$

while the measurement model is written directly in the HR coefficient space as

23 $$\begin{aligned} \tilde{\boldsymbol{\psi }}^{\textrm{HR}}_k = \boldsymbol{\psi }^{\textrm{HR}}_k + \boldsymbol{\eta }_k\,, \end{aligned}$$

where $\boldsymbol{\eta }_k$ is a zero-mean noise term with covariance $\textbf{R}_{\textrm{LSE}}$.

Because the LSE output already provides an estimate in the reduced HR space, no additional measurement operator that provides a mapping between the LR and HR space is needed, and the Kalman filter acts directly on the mismatch between the model prediction and the LSE-based estimate. The Kalman gain, therefore, simplifies to

24 $$\begin{aligned} \textbf{K}_k = \textbf{P}_{k|k-1} \left( \textbf{P}_{k|k-1} + \textbf{R}_{\textrm{LSE}} \right) ^{-1} \,, \end{aligned}$$

where $\textbf{P}_{k|k-1}$ denotes the predicted state-error covariance matrix. The corrected estimate can then be written as

25 $$\begin{aligned} \begin{aligned} \hat{\boldsymbol{\psi }}^{\textrm{HR}}_{k|k}&= \textbf{F}\,\hat{\boldsymbol{\psi }}^{\textrm{HR}}_{k-1|k-1} + \textbf{K}_k \left( \textbf{M}\,\boldsymbol{\psi }^{\textrm{LR}}_k\, - \textbf{F}\,\hat{\boldsymbol{\psi }}^{\textrm{HR}}_{k-1|k-1} \right) \,, \end{aligned} \end{aligned}$$

which highlights that the updated state is obtained by correcting the model prediction through the discrepancy between the instantaneous LSE estimate and the predicted reduced HR state. The associated covariance matrices are updated according to the standard Kalman filter recursion.

The process noise covariance is again estimated from Eq. (14), whereas the measurement noise covariance is estimated from the error between the LSE estimate and the reference HR coefficients on the validation set,

26 $$\begin{aligned} \begin{aligned} \textbf{R}_{\textrm{LSE}}&= \frac{1}{N_{\textrm{val}}-1} \textbf{E}_{\textrm{LSE}}\textbf{E}_{\textrm{LSE}}^\textsf{T} \,,\\ \textbf{E}_{\textrm{LSE}}&= \tilde{\textbf{A}}^{\textrm{HR}}_{\textrm{val}} - \textbf{A}^{\textrm{HR}}_{\textrm{val}}\,. \end{aligned} \end{aligned}$$

#### Method III—Variance-rescaled LSE-based estimation

The third method extends Method II by introducing a heuristic, mode-wise variance correction during the estimation stage. Its purpose is to compensate for the systematic attenuation of the least observable higher-order HR modes, which is a known limitation of regression-based estimators (Ewing and Citriniti 1999; Tinney et al. 2006). The correction is identified offline on the training set and then applied during the online stage. This method is hereinafter referred to as *LSE+VR*.

A diagonal rescaling matrix $\boldsymbol{\Gamma } \in \mathbb {R}^{r \times r}$ is introduced as

27 $$\begin{aligned} \boldsymbol{\Gamma } = \text {diag}\left( \left( \textbf{A}^{\textrm{HR}}_{\textrm{train}} \textbf{A}^{\textrm{HR}^{\scriptstyle \textsf{T}}}_{\textrm{train}} \right) \oslash \left( \tilde{\textbf{A}}^{\textrm{HR}}_{\textrm{train}} \tilde{\textbf{A}}^{\textrm{HR}^{\scriptstyle \textsf{T}}}_{\textrm{train}} \right) \right) ^{\circ \frac{1}{2}} \,, \end{aligned}$$

where $\text {diag}(\cdot )$ retains only the diagonal entries of its argument as a diagonal matrix, ⊘ indicates the Hadamard elementwise division, and the $\circ \frac{1}{2}$ superscript indicates the elementwise Hadamard square root. Each diagonal entry of $\boldsymbol{\Gamma }$ then equals the ratio of the empirical standard deviation of the corresponding true HR coefficients to that of its LSE estimate over the training set: multiplying the prediction by $\boldsymbol{\Gamma }$ restores the correct variance level on average, hence the name *variance rescaling*.

Accordingly, the intermediate estimate entering the Kalman correction is redefined as

28 $$\begin{aligned} \tilde{\boldsymbol{\psi }}^{\textrm{HR}}_k = \boldsymbol{\Gamma }\,\textbf{M}\,\boldsymbol{\psi }^{\textrm{LR}}_k\,. \end{aligned}$$

Method III therefore retains the same dynamical model, Kalman gain, and state-update equations introduced in Method II, the only difference being the use of the variance-rescaled intermediate estimate in place of the standard LSE prediction.

The process noise covariance is again estimated from Eq. (14), whereas the measurement noise covariance is obtained from the error between the variance-rescaled estimates and the reference HR coefficients on the validation set,

29 $$\begin{aligned} \begin{aligned} \textbf{R}_{\textrm{VR}}&= \frac{1}{N_{\textrm{val}}-1} \textbf{E}_{\textrm{VR}}\textbf{E}_{\textrm{VR}}^\textsf{T} \,,\\ \textbf{E}_{\textrm{VR}}&= \tilde{\textbf{A}}^{\textrm{HR}}_{\textrm{val}} - \textbf{A}^{\textrm{HR}}_{\textrm{val}}\,. \end{aligned} \end{aligned}$$

### HR field reconstruction and baseline comparison

**Fig. 2:**
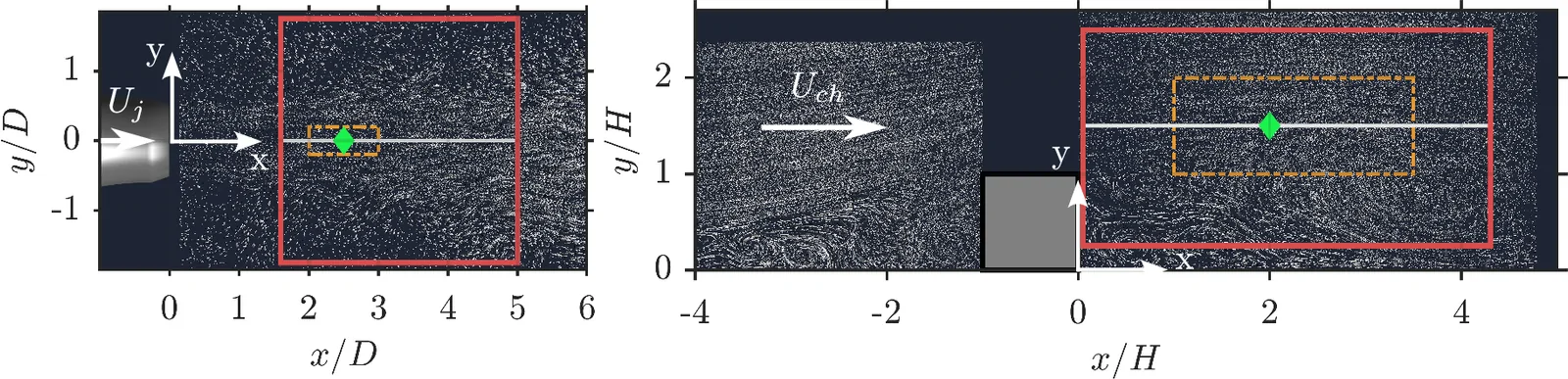
Representative multi-exposed EBV pseudo-snapshots of the two experimental configurations considered in the present work: submerged round jet (left) and channel flow over a square rib (right). The images are formed by accumulating four consecutive pulses for the jet and ten consecutive pulses for the rib case, in order to enhance the visualization of the flow structures. The so-constructed images are not used for the data processing described in Sec 3.4. The red rectangle marks the resolved region retained for the present study; the orange dashed rectangle identifies the subdomain used for the temporal premultiplied PSD analysis; the green marker denotes the probe location used for the autocorrelation analysis; the white horizontal line indicates the sampling line adopted for the spatial PSD. For detailed sketches of the full experimental setups, the reader is referred to Franceschelli et al. (2025)

For all three methods, once the Kalman-filtered estimate $\hat{\boldsymbol{\psi }}^{\textrm{HR}}_{k|k}$ of the reduced HR coefficient vector is available, the corresponding fluctuating HR snapshot is reconstructed through the retained HR POD basis as

30 $$\begin{aligned} \hat{\textbf{z}}^{\textrm{HR}}_k = \boldsymbol{\Phi }^{\textrm{HR}}_r \boldsymbol{\Sigma }^{\textrm{HR}}_r {\hat{\boldsymbol{\psi }}^{\textrm{HR}}_{k|k}}\,, \end{aligned}$$

Throughout the paper, the tilde accent $\tilde{(\cdot )}$ denotes instantaneous (pre-filtering) estimates, such as the LSE output of Eq. (21), whereas the hat accent $\hat{(\cdot )}$ denotes the Kalman-filtered estimates; the field reconstruction is always based on the latter. The full HR velocity field is then recovered by reshaping $\hat{\textbf{z}}^{\textrm{HR}}_k$ onto the HR grid and adding $\overline{\textbf{U}}^{\textrm{HR}}$.

The proposed methods are compared against direct cubic interpolation of the LR velocity field onto the HR grid. The choice is driven by the need to perform the spatial upsampling in real time, thus preventing the use of more advanced super-resolution or data-assimilation strategies. Unlike the reduced-order estimators introduced above, cubic interpolation does not exploit either the temporal evolution of the flow or the cross-resolution relationships learned from the paired HR/LR training data. It is therefore adopted as a reference baseline to assess the benefit of the proposed data-driven approaches.

## Experimental datasets and processing pipeline

The estimation framework introduced in Sect. 2 is evaluated on two experimental turbulent flow datasets acquired via pulsed-EBIV: a submerged axisymmetric water jet and an air channel flow obstructed by a spanwise square rib. These two cases were selected as representative of broadband flows, in which energy is distributed over a wide range of spatial and temporal scales. This makes them a challenging testbed for reduced-order estimation and cross-resolution reconstruction.

The pulsed-EBIV principle adopted here follows the approach introduced by Willert (2023). The channel-flow dataset is based on the experimental campaigns documented in Franceschelli et al. (2025), while the jet-flow one is obtained from an upgraded facility compared to the one described in the same work, but with similar optical arrangement. Both the setups are summarized here for completeness, together with the specific processing choices adopted in the present work. In both configurations, the coordinate system shown in Fig. 2 is adopted, with *x* and *y* denoting the streamwise and transverse directions, respectively. The main parameters of the two experimental configurations and of the corresponding LR and HR processing pipelines are summarized in Table 1.

**Table 1:** Summary of experimental and processing parameters for the two flow configurations

Parameter	Jet flow	Channel flow
Medium	Water	Air
Characteristic length	$D = 30\,\textrm{mm}$	$H = 8.18\,\textrm{mm}$
Reference velocity $U_\textrm{ref}$	$0.2\,\mathrm {m/s}$	$2.8\,\mathrm {m/s}$
Reynolds number	$Re_D \approx 6\,000$	$Re_H \approx 1\,500$
Acquisition rate	$100\,\textrm{Hz}$	$5\,\textrm{kHz}$
Laser pulse width	$1\,\textrm{ms}$	$< 50\,\textrm{ns}$
EBV camera	Prophesee EVK4 (Sony IMX636)	
Spatial resolution [pixel/mm]	≈6.5	≈30.3
Pixel displacement corresponding to $U_{ref}$ [pixel/pseudo-frame]	≈13.0	≈17.0
*LR processing (real-time pipeline)*		
Algorithm	Single-pass cross-correlation	
IW size [pixel]	48×48	64×64
Overlap	0%	
Window deformation	No	
*HR processing (offline reference)*		
Algorithm	Multi-frame, multi-pass cross-correlation	
Temporal stencil	3 frames	5 frames
IW size [pixel]	32×32	64×64
Overlap	75%	
Window deformation	Yes	
HR/LR vector/spacing ratio	6	4
Total snapshots $N_s$	9000	

### Submerged water jet

The first dataset corresponds to a submerged round jet issuing horizontally into a water tank of dimensions $900 \times 500 \times 450$ mm$^3$, corresponding to the tank length, width, and height, aligned with the *x*-, *z*-, and *y*-directions, respectively. The nozzle exit diameter is D=30 mm, and the bulk exit velocity is approximately $U_j = 0.2$ m/s, yielding a Reynolds number of $\textrm{Re}_D = U_j D / \nu \approx 6000$, with ν being the kinematic viscosity. The tank is seeded with polyamide tracer particles of nominal diameter $56\,\mu$m.

The measurement plane, passing through the jet axis, is illuminated by a pulsed laser sheet generated by a low-cost continuous-wave diode laser originally designed for engraving applications (LaserTree LT-40W-AA, with $5\,\textrm{W}$ optical power), operated in pulse-width-modulated mode. The light sheet thickness is below 2 mm at the measurement location, as estimated by imaging the sheet cross section on a calibration target during the optical alignment. The pulse repetition rate is $f_{j}=100$ Hz, with a pulse width of 1 ms.

Data are recorded using a Prophesee EVK4 event-based camera equipped with a Sony IMX636 sensor featuring a resolution of 1280×720 pixels and a pixel pitch of $4.86\,\mu \textrm{m}$. The optical magnification yields a spatial resolution of approximately 6.5 pixel/mm over a field of view of about 190×110 mm$^2$. The resolved area considered in the present study spans $1.6 \le x/D \le 5$ in the streamwise direction and $-1.75 \le y/D \le 1.75$ in the radial direction. The acquired dataset consists of 9000 velocity snapshots.

A representative multi-exposed EBV pseudo-snapshot of the jet configuration is shown in Fig. 2. For visual clarity, the image is formed here by combining four consecutive laser pulses. The figure also reports the spatial domains and sampling locations used in the analyses presented later in this work, including the resolved region, the subdomain adopted for the temporal premultiplied Power Spectral Density (PSD), the probe location used for the autocorrelation analysis, and the sampling line used for the spatial PSD. For a detailed sketch of the complete experimental setup, the reader is referred to Franceschelli et al. (2025).

### Channel flow over a square rib

The channel-flow dataset is derived from the experimental campaign described by Franceschelli et al. (2025), to which the reader is referred for full details of the facility, optical arrangement, seeding system, and camera calibration procedure. Briefly, a spanwise square rib of height $H = 8.18\,\textrm{mm}$ is mounted on the lower wall of a square-section channel of width $W = 76\,\textrm{mm}$. The free-stream velocity is $U_{ch} \approx 2.8\,\mathrm {m/s}$, corresponding to a rib Reynolds number of $\textrm{Re}_H \approx 1500$. The flow is seeded with paraffin droplets of nominal diameter $1\,\mu \textrm{m}$, injected into the upstream settling chamber, and illuminated by a high-speed pulsed Nd:YVO4 laser (Innolas/Iradion Nanio Air 532-10-V-SP). The laser pulse duration is $\sim 50\,\textrm{ns}$ and the repetition rate is $f_{ch} = 5\,\textrm{kHz}$. EBV data are acquired using the same Prophesee EVK4 camera employed for the jet experiment, with a spatial resolution of $30.3\,\mathrm {pixel/mm}$ over a field of view of approximately $42\,\textrm{mm} \times 24\,\textrm{mm}$, corresponding to about 5H×3H.

In the present work, only the wake region downstream of the rib is considered, covering $0 \le X/H \le 4.25$ in the streamwise direction and $0.25 \le Y/H \le 2.25$ in the wall-normal direction, with the origin located at the intersection between the channel wall and the rear-facing side of the rib. This region includes the separated shear layer, the recirculation bubble, the reattachment zone, and the initial downstream recovery, thus encompassing the most dynamically active portion of the flow.

The complete dataset contains 30000 velocity snapshots. In the present study, a contiguous subset of 9000 snapshots is retained to ensure consistency with the jet dataset.

A representative multi-exposed EBV pseudo-snapshot of the rib configuration is also reported in Fig. 2. In this case, the visualization is formed by combining ten consecutive laser pulses in order to better highlight the wake structure. As for the jet case, the figure identifies the resolved domain retained for the present study together with the subdomain and sampling locations used for the spectral and correlation analyses discussed in Sect. 4.

### EBV camera data processing

The raw asynchronous event stream is partitioned into pseudo-snapshots following the pulsed-EBIV procedure introduced by Willert (2023). Each laser pulse generates a burst of positive-polarity events as the illuminated tracer particles cross the per-pixel contrast threshold of the EBV sensor. The laser trigger and the event stream are synchronized through a common TTL signal, allowing the accumulation of all positive events associated with a given laser pulse over a user-defined accumulation time. A pseudo-image is therefore obtained by accumulating all positive events registered within a prescribed temporal window starting from the onset of each laser pulse. The resulting images are binary: pixels that register at least one event are assigned a value of unity, whereas pixels with no activation are assigned zero.

For both experimental cases, the binary pseudo-images are subsequently smoothed with a Gaussian kernel of standard deviation $0.75\,\textrm{pixel}$ prior to cross-correlation processing. In addition, the internal bias settings of the EVK4 camera were adjusted to suppress negative-polarity events, thereby maximizing the available bandwidth for positive events and reducing the risk of arbiter saturation.

### Low- and high-resolution EBIV processing

The same sequence of EBIV pseudo-snapshots is processed in two different ways: a computationally lightweight LR pipeline representative of a realistic real-time implementation, and a more accurate HR pipeline employed offline to generate the reference data used for training and validation.

The LR velocity fields are obtained by single-pass cross-correlation with a fixed interrogation window, without window deformation or iterative refinement. Since only one correlation step is performed for each window pair, this procedure is representative of the computational budget available in a real-time implementation. No additional post-processing is applied apart from the universal outlier detection (Westerweel and Scarano 2005).

The HR velocity fields are instead computed using an in-house multi-frame, multi-pass cross-correlation algorithm. At each time step, a stencil of consecutive pseudo-snapshots is used to improve the quality of the correlation peak, which is particularly beneficial for EBIV data, where individual pseudo-images are binary and contain limited intensity information. The resulting HR fields provide a spatially refined and temporally regularized reference that the estimator is trained to recover from the corresponding LR measurements.

Table 1 reports the interrogation window sizes, overlaps and temporal stencil dimensions adopted for each dataset in both processing ways.

A remark is due on the different interrogation window overlap adopted in the two pipelines (0% for LR, 75% for HR). Overlapping windows increase the vector density but not, per se, the spatial resolution: cross-correlation over a window of linear size $L_{IW}$ acts as a spatial moving-average filter on the underlying displacement field, with a frequency response set by the window size and by the selected weighting window (Astarita 2007). Velocity fluctuations of streamwise wavelength $\lambda _x$ are thus increasingly attenuated as $\lambda _x$ approaches $L_{IW}$ and can be considered well-resolved only for $\lambda _x \gtrsim 2 L_{IW}$; moreover, neighbouring HR vectors share particle-image information and exhibit a processing-induced spatial correlation over distances of the order of $L_{IW}$, regardless of the overlap. For the present HR processing, this bound corresponds to well-resolved content for $D/\lambda _x \lesssim 3$ in the jet case and $H/\lambda _x \lesssim 2$ in the channel-flow case. The actual role of the 75% overlap is to sample this window-filtered field without aliasing: at 0% overlap the sampling spacing equals the filter size itself, so that the attenuated but non-negligible content in the band $L_{IW} \lesssim \lambda _x \lesssim 2L_{IW}$ is aliased, whereas the fourfold oversampling represents it cleanly. The genuine gains of the HR pipeline over the LR one come instead from a combination of the smaller interrogation window (although not strictly required), the window deformation, which improves the spectral response towards the filter bound (Scarano 2002; Astarita 2007), and the multi-frame temporal stencil, which improves the correlation signal-to-noise ratio. Notably, for the channel-flow case the two pipelines share the same window size, so that the HR reference mainly provides denser, alias-free sampling and higher fidelity rather than a wider resolved wavenumber range. The learned LR-to-HR mapping therefore targets the HR representation as produced by the offline pipeline, including its correlation structure: the estimators recover physically independent fine-scale content only up to the effective spatial resolution of the HR processing and reproduce its smoothing characteristics beyond that limit. This should be kept in mind when interpreting the spectral analyses of Sect. 4.4, where the recovered small-scale content is bounded by the resolution of the HR reference itself.

The datasets are then divided into training, validation, and testing subsets. The training subset is used to construct the reduced-order spaces and identify the mapping operators, the validation subset is used to estimate the Kalman filter noise statistics (Sect. 2.2.2), and the testing subset is reserved for the final performance assessment. The dataset is split into 4500 snapshots for training, 1500 for validation, and 2000 for testing, with two buffer regions of 500 snapshots inserted between consecutive subsets to reduce temporal correlation.

## Results

**Fig. 3:**
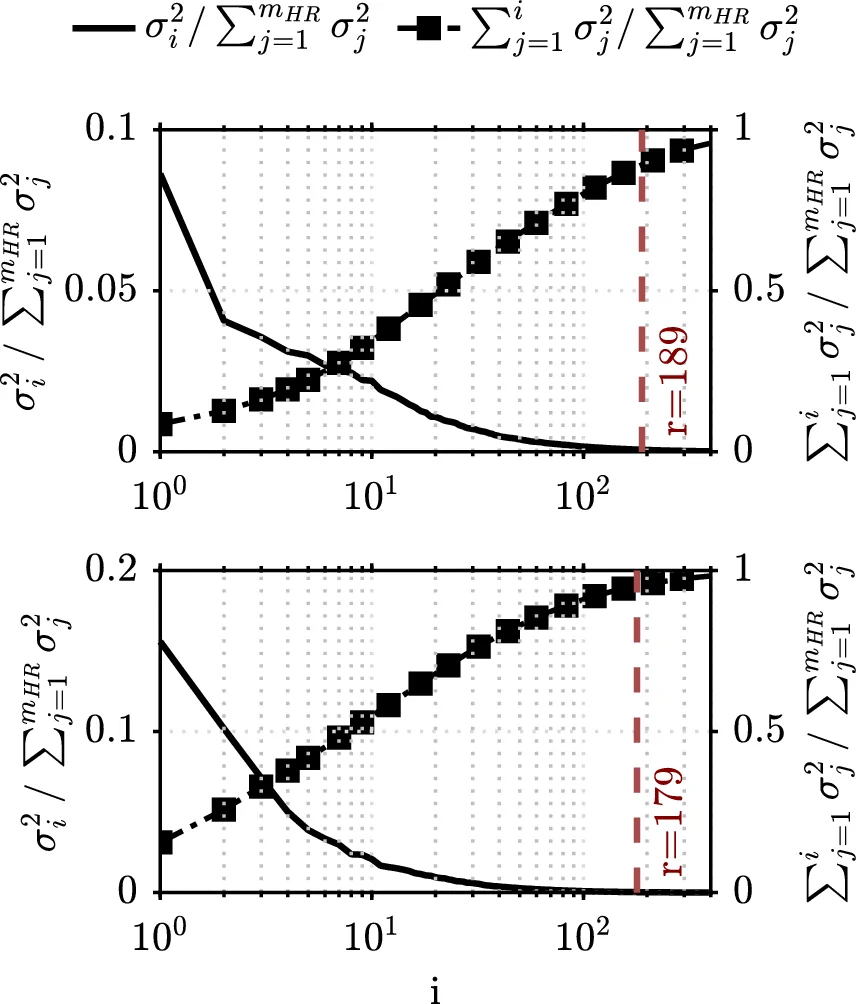
POD modal energy spectrum and cumulative POD energy for the two considered HR datasets: top, jet flow; bottom, channel flow. The solid black line refers to the left vertical axis and reports the normalized contribution of the *i*-th mode, $\sigma _i^2 / \sum _{j=1}^{m} \sigma _j^2$. The black dashed line with square markers refers to the right vertical axis and reports the corresponding cumulative contribution, $\sum _{j=1}^{i} \sigma _j^2 / \sum _{j=1}^{m} \sigma _j^2$. The red vertical dashed line indicates the truncation rank *r* selected by the elbow criterion

**Fig. 4:**
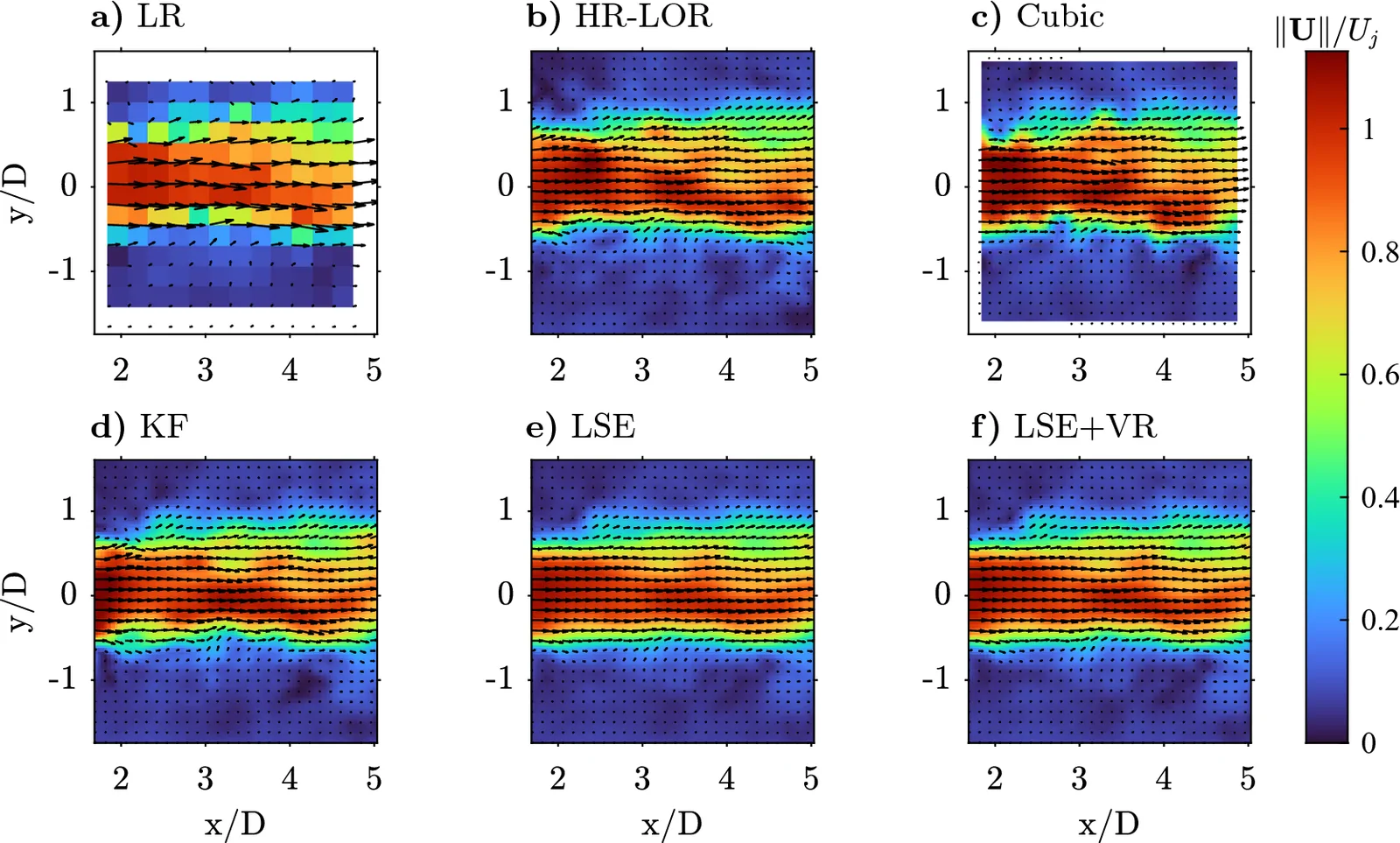
Comparison of instantaneous velocity field realizations for the jet-flow case. Panels show: **a** input LR field; **b** reference HR-LOR field; **c** HR field obtained by direct cubic interpolation of the LR measurement; **d** HR estimate obtained with KF; **e** HR estimate obtained with LSE; and **f** HR estimate obtained with LSE+VR. Colour contours represent the instantaneous velocity magnitude $\Vert \textbf{U}\Vert$, normalized by the jet bulk velocity $U_j$, while arrows indicate the in-plane velocity vectors. For readability, one vector every three is shown in the HR panels

**Fig. 5:**
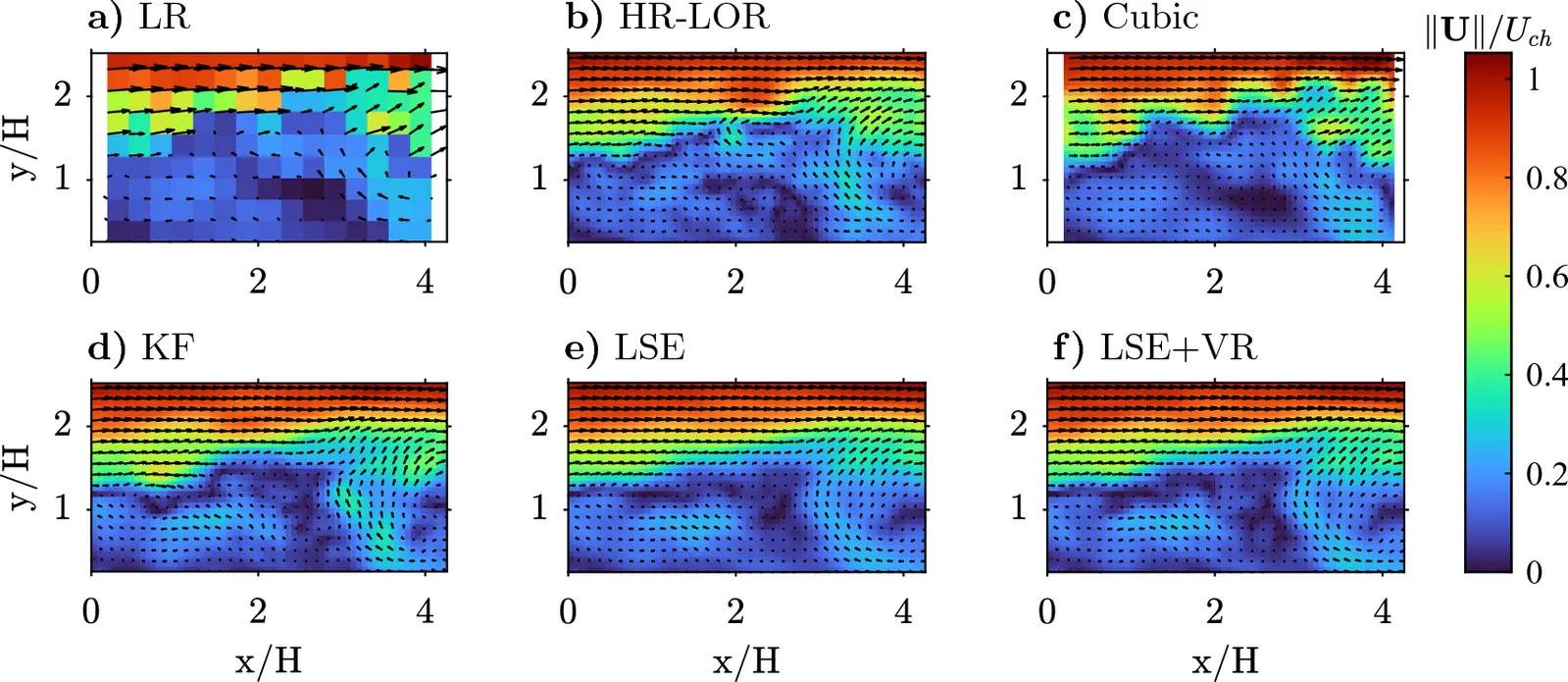
Comparison of instantaneous velocity field realizations for the channel-flow case. Panels show: **a** input LR field; **b** reference HR-LOR field; **c** HR field obtained by direct cubic interpolation of the LR measurement; **d** HR estimate obtained with KF; **e** HR estimate obtained with LSE; and **f** HR estimate obtained with LSE+VR. Colour contours represent the instantaneous velocity magnitude $\Vert \textbf{U}\Vert$, normalized by the free-stream channel-flow velocity $U_{ch}$, while arrows indicate the in-plane velocity vectors. For readability, one vector every two is shown in the HR panels

This section evaluates the three proposed estimation strategies on the jet and channel-flow datasets. The discussion is organized hierarchically, from instantaneous field reconstruction to increasingly demanding statistical and spectral diagnostics. Throughout the section, the reference truth used for quality metrics is assumed to be the HR low-order reconstruction (referred in short as HR-LOR), obtained by projecting the HR dataset onto the truncated POD basis introduced in Sect. 2. The three estimators (KF, LSE, and LSE+VR) are compared against this reference and against direct cubic interpolation of the LR field onto the HR grid, which is adopted as a purely spatial, non-model-based baseline.

It must be stressed that the HR-LOR reference is not an independent ground truth. Rather, it is the low-order projection of the HR fields obtained from the same event stream through the offline processing pipeline. The reported errors therefore quantify how closely the real-time estimators recover the offline-grade reduced-order representation, rather than their absolute deviation from the true flow state. This is consistent with the objective of the present framework, which is precisely to make an offline-grade representation available at real-time rates. The fidelity of this target with respect to conventional velocimetry has, moreover, already been established: in Franceschelli et al. (2025), pulsed-EBIV measurements on the same flow configurations were benchmarked against synchronized, simultaneous PIV measurements—which can fairly be regarded as the reference—with the low-order reconstructions deviating from their PIV counterparts by errors of the order of 0.5% of the peak turbulent kinetic energy for the jet flow, and a few times higher for the channel flow at the truncation ranks of interest here.

### POD modal energy distribution and retained energy

The resulting POD modal energy distributions for the two HR datasets are reported in Fig. 3. Since the energy captured by the *i*-th POD mode within the dataset equals the square of the corresponding singular value, $\sigma _i^2$, the singular value distribution coincides with the POD modal energy spectrum, and its running sum with the cumulative retained energy. For each case, the figure shows the normalized modal energy $\sigma _i^2$ together with its cumulative contribution as functions of the mode index *i*. The red vertical line indicates the truncation rank selected by the *elbow criterion* introduced above. The adopted threshold yields r=189 for the jet case and r=179 for the channel-flow case, corresponding to retained cumulative energies of 89.1% and 95.2%, respectively. In both datasets, the singular value decay is gradual rather than characterized by a sharp cut-off. As a result, the selected truncation lies in the high-order portion of the spectrum while still retaining most of the resolved modal energy. This behaviour highlights the broadband character of the two flow cases and the wide distribution of energy across spatial scales, making these datasets particularly challenging for reduced-order reconstruction and estimation.

### Instantaneous field reconstruction

A first qualitative assessment is presented in Figs. 4 and 5 for the jet and channel-flow cases, respectively, depicting the instantaneous velocity fields. Each figure compares the input LR field (a), the HR-LOR reference (b), the field obtained by direct cubic interpolation (c), and the three reconstructed fields obtained with KF (d), LSE (e), and LSE+VR (f). The colour contours represent the instantaneous velocity magnitude $\Vert \textbf{U}\Vert$, while the quiver plot indicates the in-plane velocity vector. The LR field provides only a coarse and noisy representation of the instantaneous flow, whereas the HR-LOR field reveals substantially finer spatial detail, including sharp velocity gradients and the coherent structures that characterize each flow: the shear layers and vortical roll-up of the jet core, and the separated region downstream of the square rib in the channel-flow case. All three proposed estimators recover the broad spatial organization of the HR-LOR field with good fidelity. The KF estimate provides a smooth reconstruction that closely follows the large-scale coherent features of the reference. The LSE reconstruction captures the same dominant structures, although the smallest-scale features appear less sharply defined. The LSE+VR reconstruction recovers additional small-scale content while maintaining spatial regularity. In contrast, the cubic-interpolation baseline introduces visible high-frequency oscillations and artificial spatial modulations that are absent in the reference field. These artefacts are particularly evident in the channel-flow case, where the interpolation of the noisy LR measurements produces spurious patterns in regions of strong velocity gradient.

### Statistics of the reconstructed fields

**Fig. 6:**
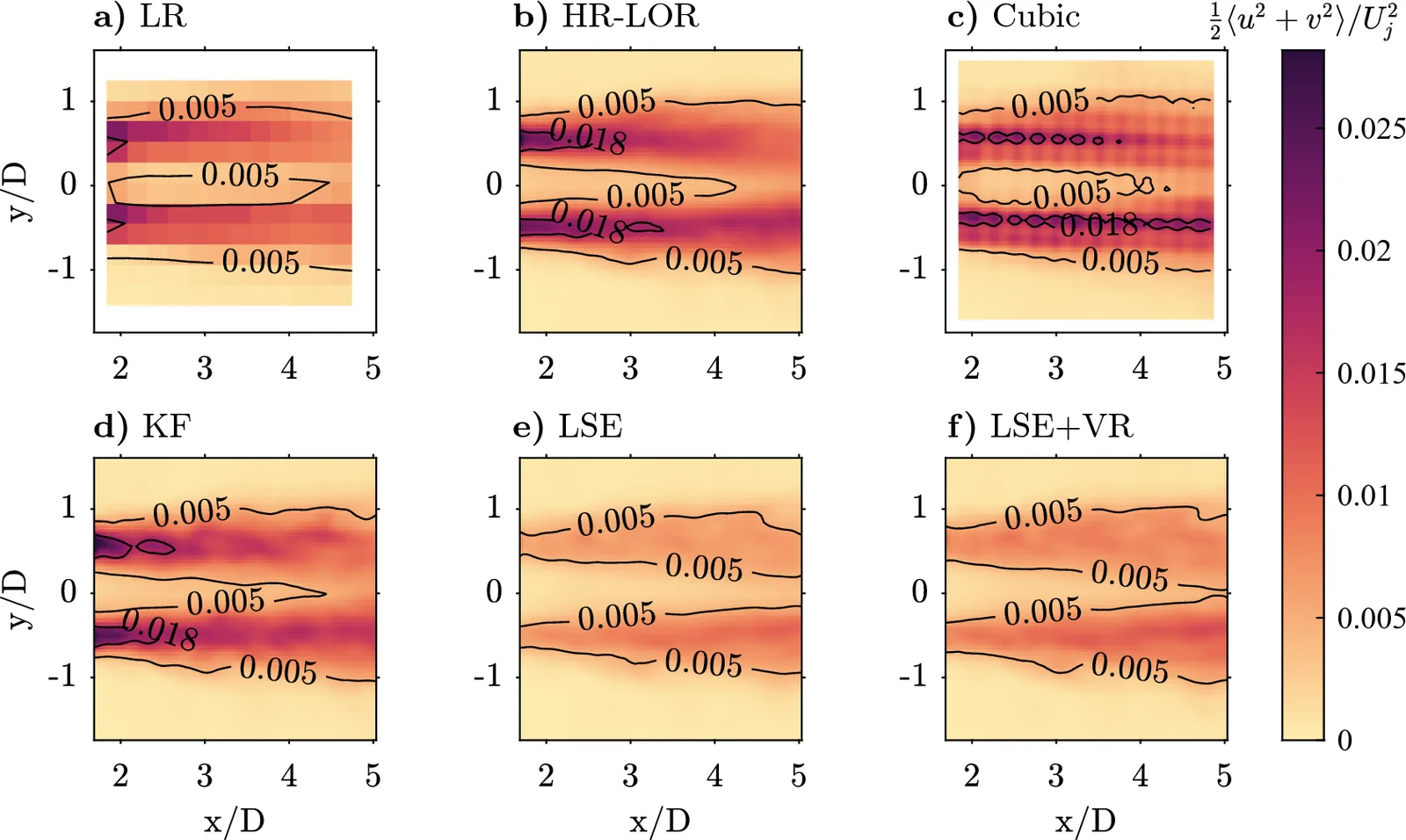
Spatial distribution of TKE for the jet-flow case, normalized by the square of the jet bulk velocity $U_{j}$. Panels show: **a** LR input; **b** HR-LOR reference; **c** cubic interpolation; **d** KF; **e** LSE; and **f** LSE+VR. Black isolines denote selected TKE levels

**Fig. 7:**
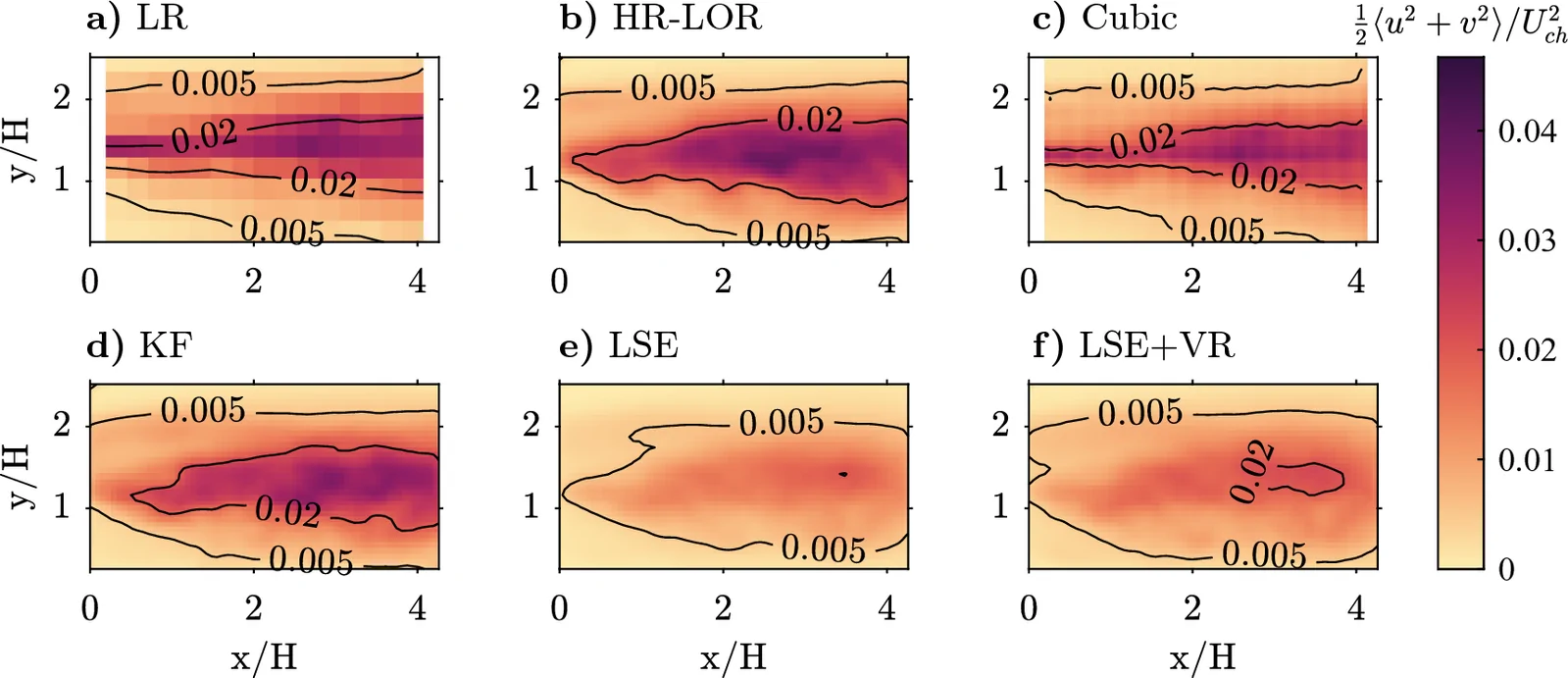
Spatial distribution of TKE for the channel-flow case, normalized by the square of the free-stream channel-flow velocity $U_{ch}$. Panels show: **a** LR input; **b** HR-LOR reference; **c** cubic interpolation; **d** KF; **e** LSE; and **f** LSE+VR. Black isolines denote selected TKE levels

The spatial distribution of the in-plane Turbulent Kinetic Energy (TKE), defined here as $\frac{1}{2}\langle u^2(\textbf{x},t)+v^2(\textbf{x},t)\rangle$, is reported in Figs. 6 and 7 for the jet and channel-flow cases, respectively.

The HR-LOR reference reveals clear and physically consistent high-energy regions. In the jet, the TKE is concentrated along the two shear layers, where the strongest fluctuations are associated with the roll-up and downstream convection of coherent structures. In the channel flow, the highest energy levels are found in the separated shear layer and in the downstream recovery region.

The three proposed estimators recover these energetic regions with good overall consistency. Among them, the KF result is the closest to the reference in both spatial footprint and amplitude, with high-energy regions appearing at the correct locations and with a realistic spatial spreading. The LSE reconstruction also captures the overall topology of the TKE field, but the energetic regions appear smoother and less sharply defined, with reduced peak levels, indicating an attenuation of the turbulent fluctuations. This behaviour is consistent with the tendency of linear regression estimators to smooth the fluctuating content, especially for less energetic and less correlated components of the flow. The LSE+VR correction mitigates this effect by restoring part of the missing modal variance, leading to a better recovery of the energy distribution while preserving a physically regular spatial organization.

The cubic-interpolation baseline performs substantially worse in both cases. The reconstructed field exhibits periodic energy modulations that are absent in the reference, visible as alternating bands or ripples of higher and lower TKE, particularly along the jet shear layers and in the energetic region of the channel flow. These artefacts arise from grid imprinting: when under-resolved and noisy LR fluctuating fields are interpolated onto a finer grid, the interpolation operator transfers the footprint of the original sampling pattern into the reconstructed field. Because TKE is a quadratic quantity, these non-physical oscillations are amplified and become much more evident than in the instantaneous velocity snapshots.

Overall, these results show that the main benefit of the proposed estimators is not limited to reconstructing plausible instantaneous fields, but extends to recovering fluctuation statistics with a physically consistent spatial organization and without the grid-imprinting artefacts observed with interpolation.

### Temporal and spatial spectral content

The spectral fidelity of the reconstructed fields is assessed through pre-multiplied temporal and spatial spectra of the streamwise velocity fluctuation *u*. In the following, $\langle \cdot \rangle _T$ denotes temporal averaging and $\langle \cdot \rangle _{\Omega }$ denotes spatial averaging over the spatial subdomain Ω.

**Fig. 8:**
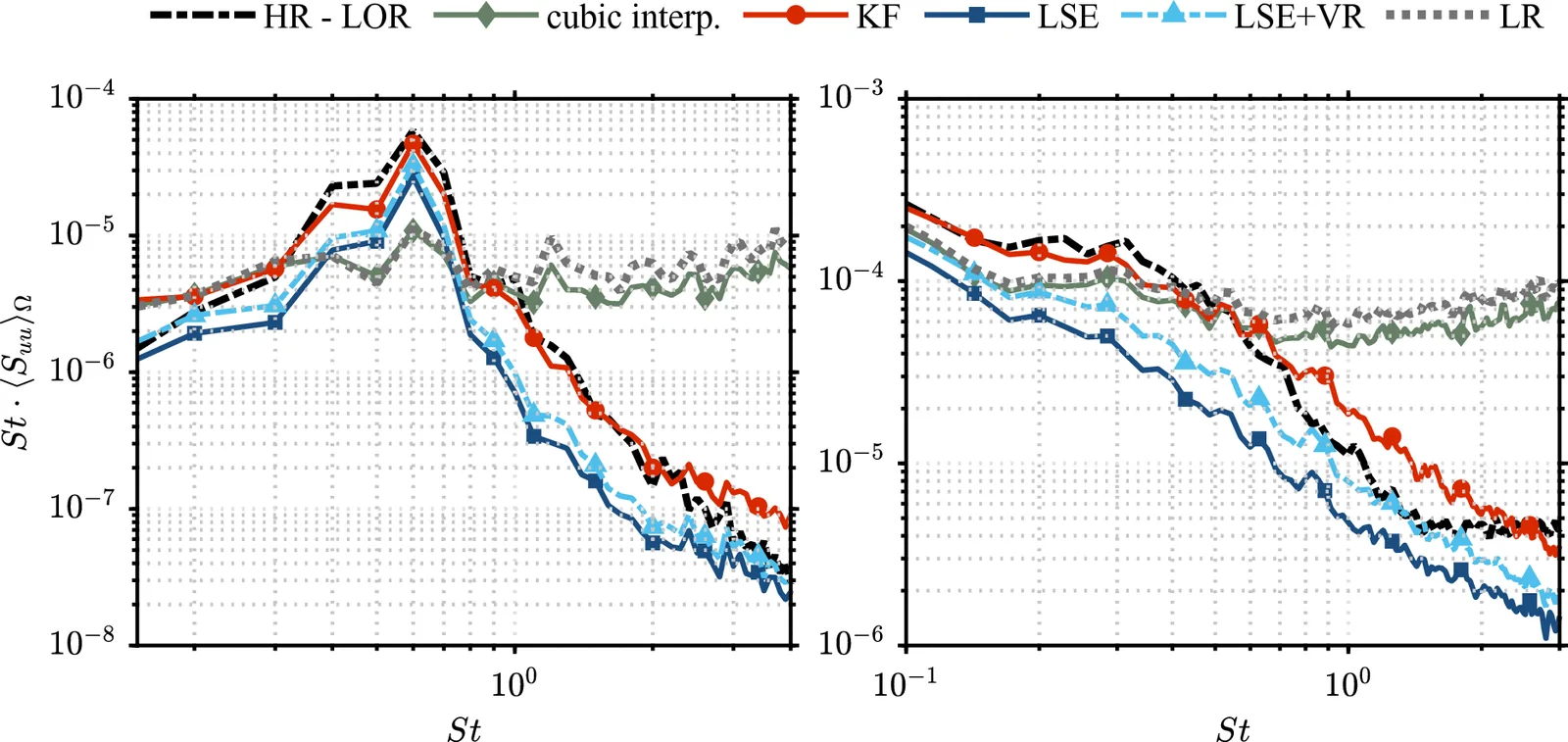
Premultiplied temporal PSDs of the streamwise velocity fluctuations for the jet (left) and channel-flow (right) cases, normalized, respectively, by $U_j$ and $U_{ch}$, reported as $St\,\langle S_{uu}\rangle _{\Omega }$. The spectra are spatially averaged over the subdomain Ω defined by $2 \le x/D \le 3$ and $-0.2 \le y/D \le 0.2$ for the jet, and $1 \le x/H \le 3.5$ and $1 \le y/H \le 2$ for the channel flow, and compared against the HR-LOR reference. The premultiplied spectra are reported in non-dimensional form, with the spectral energy normalized by $U_j^2$ and $U_{ch}^2$, respectively. The grey dotted line reports the spectra of the raw LR fields

The quantity reported in the temporal spectral analysis of Fig. 8 is the premultiplied temporal PSD of the streamwise velocity fluctuation, $St\,\cdot \langle S_{uu}\rangle _{\Omega }$, computed from the local time signals within the subdomain Ω, and the Strouhal number is defined as $St=fD/U_j$ for the jet and $St=fH/U_{ch}$ for the channel flow. The temporal spectra are then averaged over Ω, defined as $2 \le x/D \le 3$ and $-0.2 \le y/D \le 0.2$ for the jet, and $1 \le x/H \le 3.5$ and $1 \le y/H \le 2$ for the channel flow, corresponding, respectively, to the jet core and the highly turbulent region behind the square rib. The corresponding subdomains are indicated by the orange dashed rectangles in Fig. 2.

The jet subdomain covers a comparatively compact region of size 1D×0.4D, centred on the jet core, where the signature of the jet-column coherent dynamics emerges most clearly and the comparison between estimators is less affected by measurement noise. The channel-flow subdomain is deliberately more extended: it samples a fully turbulent broadband region (representative of the behaviour that would also be observed across a turbulent shear layer) and the wider spatial averaging promotes the statistical convergence of the spectra, at the price of smoothing out strictly local contributions. It has been verified that the reported trends are insensitive to this choice: repeating the analysis over a much smaller nested subdomain leaves the relative behaviour of the estimators unchanged, at the cost of noisier spectra; the jet case trends were likewise verified by recomputing the spectra within the shear layer region. The two cases are therefore regarded as complementary: the former probes the recovery of coherent, spectrally localized dynamics, the latter the recovery of broadband turbulent content.

**Fig. 9:**
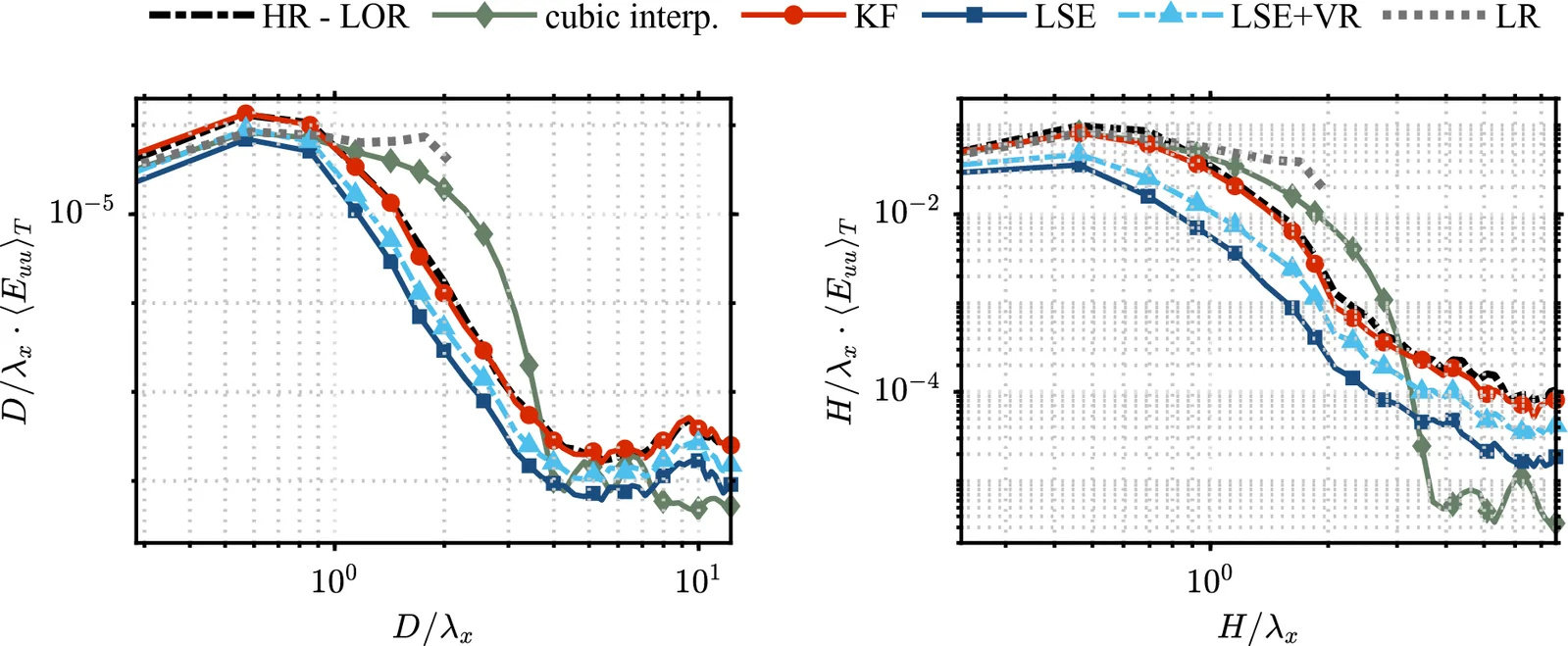
Premultiplied spatial spectra of the streamwise velocity fluctuations for the jet (left) and channel-flow (right) cases, normalized, respectively, by $U_j$ and $U_{ch}$, computed along the horizontal lines y/D=0 and y/H=1.5, respectively. The spectra are shown as $(D/\lambda _x)\,\langle E_{uu}\rangle _T$ for the jet and $(H/\lambda _x)\,\langle E_{uu}\rangle _T$ for the channel flow, and compared against the HR-LOR reference. The premultiplied spectra are reported in non-dimensional form, with the spectral energy normalized by $U_j^2$ and $U_{ch}^2$, respectively. The grey dotted line reports the spectra of the raw LR fields, whose resolved wavenumber range terminates earlier owing to the coarser LR vector spacing

The premultiplied spatial spectra, shown in Fig. 9, are computed along a horizontal sampling line located at y/D=0 for the jet and at y/H=1.5 for the channel flow, as indicated by the grey horizontal line in Fig. 2. In this case, the spectra are represented in premultiplied non-dimensional form as $(L_{ref}/\lambda _x)\,\langle E_{uu}\rangle _T$, where $E_{uu}$ denotes the one-dimensional streamwise spatial spectrum of *u*, $L_{ref}$ is a reference length (*D* for the jet and *H* for the channel flow), and $\lambda _x$ is the spatial wavelength.

The temporal spectra show that all three proposed estimators capture the dominant frequency content of the flow. In the jet case, the main spectral peak associated with the dominant coherent dynamics is recovered in both position and amplitude. In the channel-flow case, where the spectrum is broader and less sharply peaked, the low-frequency content is likewise captured correctly. The KF estimator provides the closest agreement with the HR-LOR reference over the widest frequency range, including part of the high-frequency tail, capturing higher energy content with less attenuation, consistently with the TKE results. Standard LSE and LSE+VR follow the same overall spectral shape and retain the dominant dynamics, but with a lower overall energy level, consistent with the attenuated fluctuating fields already observed in the TKE maps. The LSE+VR is observed to perform consistently better than the standard counterpart.

The cubic-interpolation baseline again exhibits the least reliable behaviour. Although it reproduces part of the low-frequency content (albeit with noticeable attenuation), it develops a markedly elevated broadband floor at intermediate and high Strouhal numbers. This excess floor is more than one order of magnitude above the reference over part of the spectrum, and in the jet tail it approaches nearly two orders of magnitude. As a result, the physical spectral decay is partially obscured and the identification of dominant peaks becomes less reliable, particularly in the channel-flow case where the broadband contamination masks part of the spectral structure. The spectra of the raw LR fields, also reported in Fig. 8, make the origin of this floor explicit: the LR spectra follow the reference at low Strouhal numbers, but flatten into an elevated broadband floor at intermediate and high frequencies, which constitutes the spectral signature of the measurement noise of the lightweight single-pass processing. The cubic-interpolation baseline essentially inherits this floor, as expected from a purely spatial operator acting on the LR fields, whereas the proposed estimators suppress it by constraining the reconstruction to the learned reduced-order subspace. These observations are consistent with the known spectral response of conventional PIV (Foucaut et al. 2004).

The spatial spectra reinforce the same interpretation from a complementary perspective. The three data-driven estimators reproduce the HR-LOR spatial energy distribution over a broad range of resolved wavelengths, including part of the small-scale content that is absent from the raw LR field. The raw LR spectra, also included in Fig. 9, follow the reference over the largest resolved scales with a slight energy excess ascribable to measurement noise, but terminate at markedly smaller wavenumbers than all the other curves: the coarse LR vector spacing bounds the shortest resolvable wavelength to the corresponding Nyquist limit, $\lambda _{x,\textrm{min}} = 2\Delta x_{\textrm{LR}}$, i.e. $D/\lambda _x \approx 2$ for the jet and $H/\lambda _x \approx 2$ for the channel flow. This early cut-off visualizes directly the spatial information gap that the proposed estimators are required to fill. The KF result provides the closest overall match and, especially in the jet case, nearly overlaps the HR-LOR reference over most of the resolved spectral range. The advantage of the VR correction is particularly evident in this representation: compared with standard LSE, LSE+VR recovers part of the missing energy while preserving the correct spectral trend. In contrast, cubic interpolation departs from the reference already at intermediate wavelengths and exhibits characteristic undulations at wavelengths comparable to the original LR vector spacing. In the jet case, this interpretation is quantitatively consistent with the LR sampling pitch. From the image scaling, the LR vector spacing is $\Delta x_{\textrm{LR}} \approx 7.4~\textrm{mm}$, corresponding to $D/\Delta x_{\textrm{LR}} \approx 4.1$, namely about four LR vectors across the nozzle diameter. The interpolated spectrum indeed departs from the reference around this scale, displaying an energy deficit (notch) near $D/\lambda _x \approx 4$ followed by a spurious energy excess at slightly smaller wavelengths, $D/\lambda _x \approx 5$–6. These undulations result directly from grid imprinting and confirm that the interpolation baseline does not reconstruct the missing HR content, but instead introduces artificial spatial scales into the field.

From a physical standpoint, these spectra show that the proposed framework not only improves the visual quality of the reconstructed fields but also preserves the scale distribution of the fluctuating dynamics. This aspect is essential if the reconstructed fields are to be used for reduced-order modelling, system identification, or feedback-oriented state estimation.

### Reduced-order dynamics

**Fig. 10:**
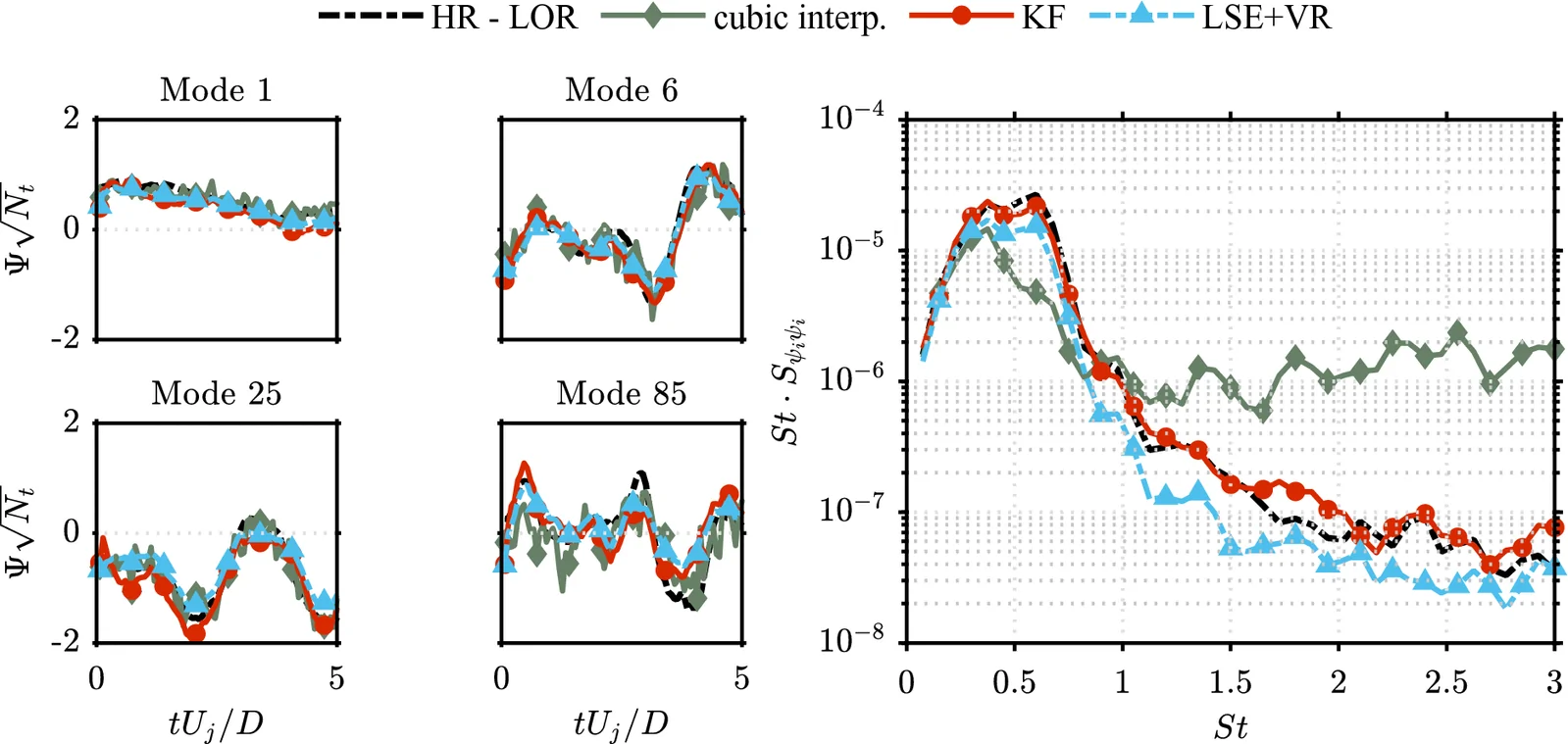
Jet-flow reduced-order dynamics. Left: time histories of selected POD coefficients (modes 1, 6, 25, and 85) over five convective time unit. Right: premultiplied spectrum of the mode-6 coefficient. Coefficients are normalized as $\psi _i/\sqrt{N_t}$. For visual clarity, only the LSE+VR result is shown among the LSE-based estimators

**Fig. 11:**
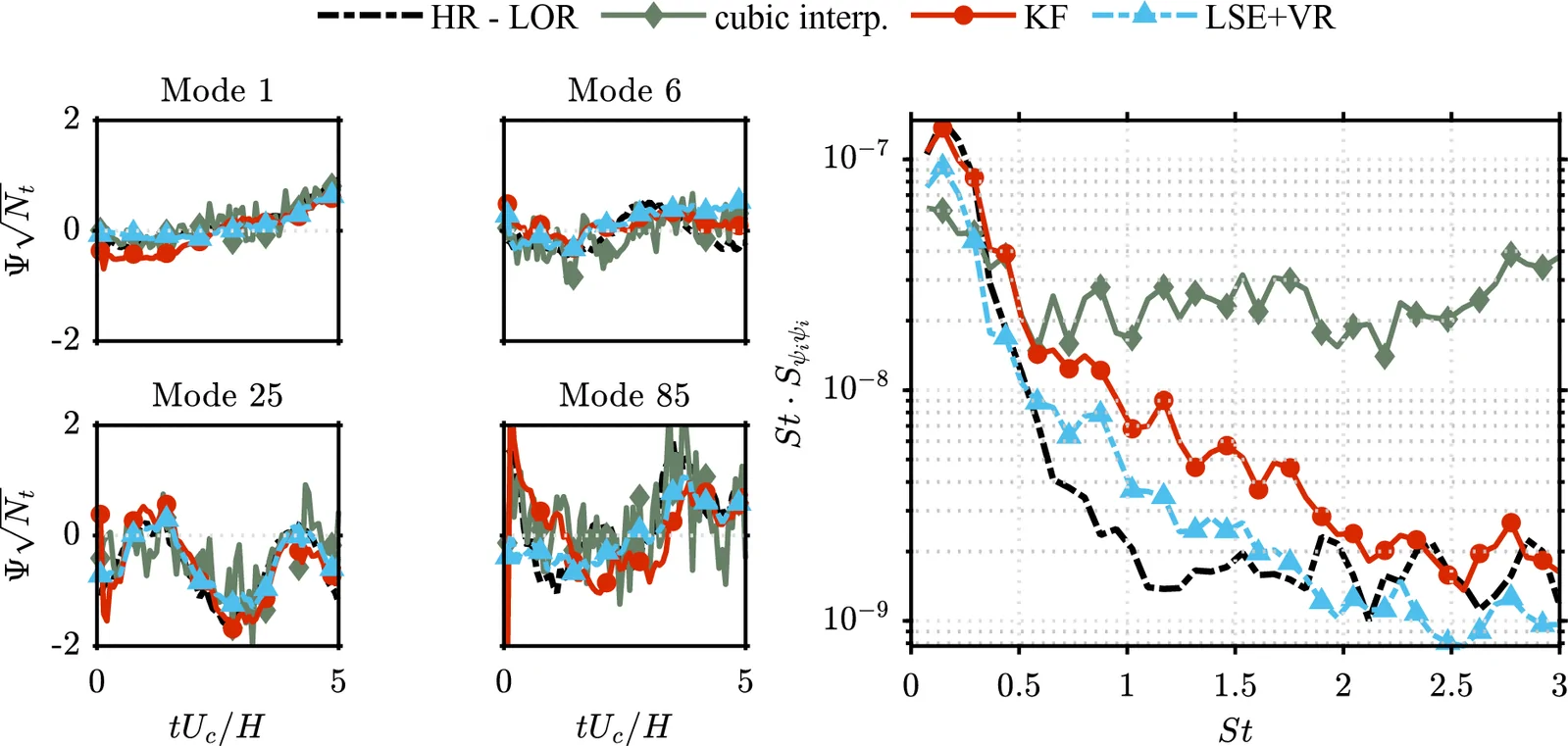
Channel-flow reduced-order dynamics. Left: time histories of selected POD coefficients (modes 1, 6, 25, and 85) over five convective time units. Right: premultiplied spectrum of the mode-6 coefficient. Coefficients are normalized as $\psi _i/\sqrt{N_t}$. For visual clarity, only the LSE+VR result is shown among the LSE-based estimators

To assess the estimation accuracy directly in the reduced space, Figs. 10 and 11 compare the time histories of four selected reduced-order coefficients together with the premultiplied spectrum of a representative one. The left-hand side of each figure reports the temporal evolution of the coefficients associated with modes 1, 6, 25, and 85 over a window of five convective time units. For each mode *i*, the plotted quantity is the corresponding scalar component of the reduced state, namely $\psi _{k,i}^{\textrm{HR}}$ for the HR-LOR reference and $\hat{\psi }_{k|k,i}^{\textrm{HR}}$ for the estimate, normalized by $\sqrt{N_t}$.

The convective time is defined as $t_c=tU_{ref}/L_{ref}$, with $U_{ref}=U_j$ and $L_{ref}=D$ for the jet, and $U_{ref}=U_{ch}$ and $L_{ref}=H$ for the channel flow. The four modes are selected to span the entire retained spectrum: the most energetic mode (mode 1), an intermediate-energy mode still dominated by coherent dynamics (mode-6), and two progressively higher-order, low-energy modes (modes 25 and 85), for which the estimation problem becomes increasingly challenging. The right-hand side shows the premultiplied spectrum of the coefficient associated with mode 6, adopted as representative example because it combines a significant energy content with a well-defined spectral signature, which makes the differences between estimators clearly visible. For visual clarity, only the LSE+VR result is shown among the LSE-based estimators, as in this representation it largely overlaps with the standard LSE result.

This analysis is particularly informative because the reduced coefficients are the actual state variables estimated by the proposed methods. Accurate coefficient reconstruction therefore implies not only a faithful reconstruction of the velocity field, but also a reliable low-dimensional representation of the underlying dynamics.

For the leading modes, i.e. the most energetic modes retrieved at the lowest mode indices, all reconstruction methods recover the overall trend of the HR-LOR reference reasonably well. The KF and LSE+VR estimates are generally the most consistent, reproducing the dominant low-order dynamics with good agreement in both amplitude and phase. Cubic interpolation often follows the large-scale trend as well, but already at low mode number, it exhibits visibly noisier coefficient traces, with superposed fluctuations that are absent from the reference. This behaviour is particularly evident in the channel-flow case, which is more strongly affected by measurement noise. As the mode rank increases, the reconstruction problem becomes progressively harder because the higher-order coefficients carry less energy and lie closer to the noise floor of the LR measurements. In this regime, the interpolation-based estimates become much less trustworthy and may develop large spurious excursions, whereas the model-based estimators retain a meaningful correlation with the reference.

The attenuation of the LSE-based reconstruction remains visible at higher mode number, as illustrated for instance by mode 85 in the channel-flow case. The VR correction partially compensates for this loss by restoring part of the missing modal variance, yielding a reconstruction with amplitudes comparable with KF. The coefficient spectra confirm the same interpretation. In the jet case, KF reproduces the reference spectrum most closely over the widest Strouhal range. In the channel-flow case, the situation partially reverses: the KF spectrum exceeds the reference at high frequencies, indicating that part of the measurement noise is transferred to the estimated coefficients through the correction step, whereas the smoother LSE+VR estimate remains in part closer to the reference over that range. This interchange in the relative performance of the two estimators between the two flow cases reflects the different role played by the linear dynamical model: in the jet, the quasi-periodic coherent dynamics are well captured by the one-step transition model, which allows the KF to filter the measurement noise effectively; in the broadband channel flow, the linear model is less informative, and the KF correction step consequently admits a larger share of the measurement noise. The same trend is observed across the other retained modes, with the KF high-frequency excess visible in the channel-flow case over a wide range of mode indices, and the LSE+VR attenuation persisting at high mode index in both flow cases. It is worth remarking that both estimators consistently reproduce the *shape* of the coefficient spectra (the spectral decay and the distribution of the dominant content) with the discrepancies essentially confined to the energy level, as in the LSE+VR attenuation. By contrast, the spectra of the cubic-interpolation baseline are nearly flat beyond the low-frequency range: the physical spectral decay is lost altogether, indicating that the interpolated coefficients are dominated by noise rather than by the underlying modal dynamics. By contrast, cubic interpolation displays a clearly elevated broadband floor, confirming that it does not simply lose information, but also injects spurious modal activity.

### Temporal coherence from virtual probes

**Fig. 12:**
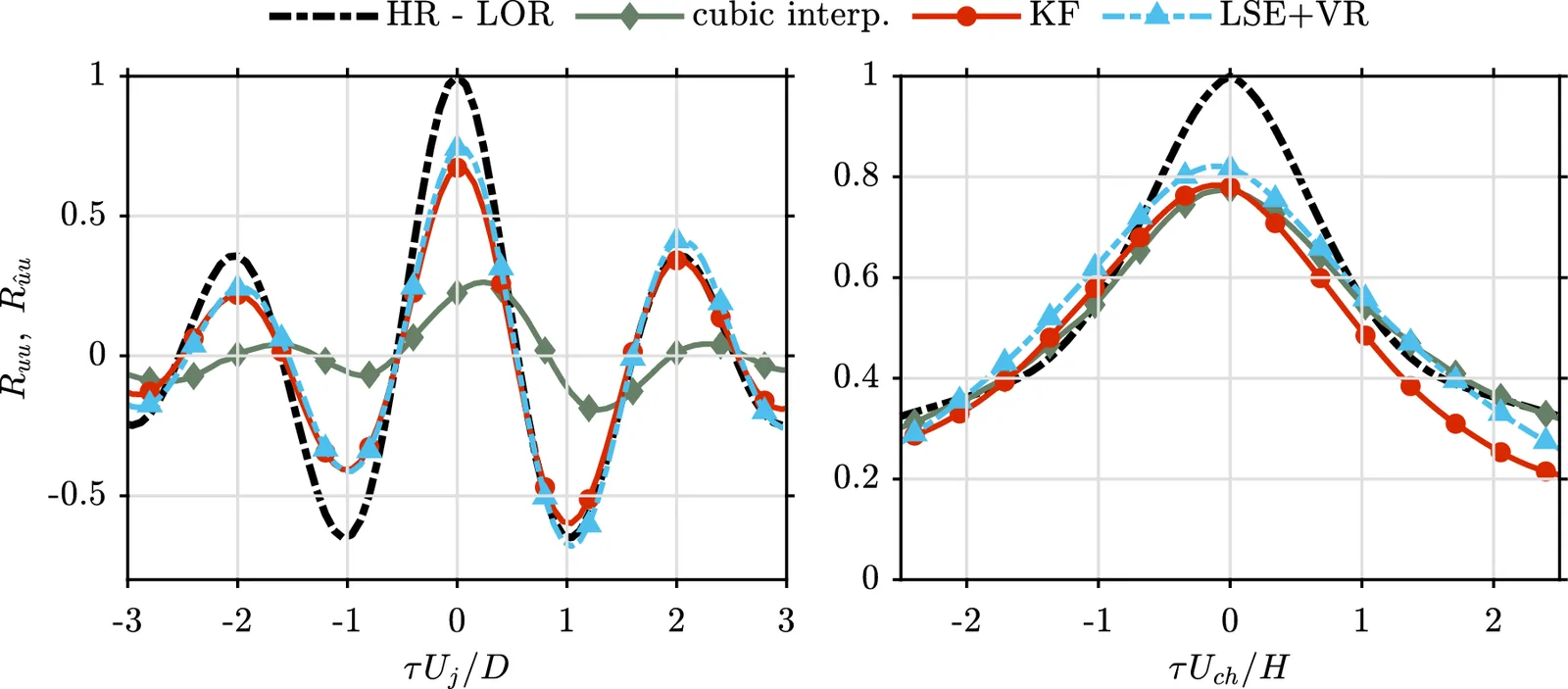
Lag-correlation analysis of the streamwise velocity fluctuation *u* from virtual probes located at (x/D,y/D)=(2,0) for the jet (left) and (x/H,y/H)=(2,1.5) for the channel flow (right): temporal autocorrelation $R_{uu}$ for the HR-LOR reference, and normalized cross-correlation with the reference, $R_{\hat{u}u}$, for the estimators. The time lag is normalized as $\tau U_j/D$ for the jet and $\tau U_{ch}/H$ for the channel flow. For visual clarity, only the LSE+VR result is shown among the LSE-based estimators

A complementary assessment of the reconstruction quality is provided by a lag-correlation analysis at a virtual probe located at (x/D,y/D)=(2,0) for the jet and (x/H,y/H)=(2,1.5) for the channel flow, respectively. For the HR-LOR reference, the temporal autocorrelation of the streamwise velocity fluctuation is reported,

31 $$\begin{aligned} R_{uu}(\tau )=\frac{\langle u(t)\,u(t+\tau )\rangle }{\langle u^2(t)\rangle }, \end{aligned}$$

which is symmetric by construction. For the estimators, the normalized cross-correlation between the estimated and the reference probe signals is shown instead,

32 $$\begin{aligned} R_{\hat{u}u}(\tau )=\frac{\langle \hat{u}(t)\,u(t+\tau )\rangle }{\sigma _{\hat{u}}\,\sigma _{u}}, \end{aligned}$$

where $\sigma _{\hat{u}}$ and $\sigma _{u}$ denote the standard deviations of the estimated and reference signals. This cross-statistic is adopted deliberately, since it is the only one able to reveal a temporal misalignment between reconstruction and reference: an artificial time lag would displace the correlation peak away from τ=0. The results are reported in Fig. 12. For visual clarity, only the LSE+VR result is shown among the LSE-based estimators.

In the jet case, the HR-LOR reference displays the expected quasi-periodic symmetric autocorrelation signature associated with the passage of coherent vortical structures and with a well-defined characteristic timescale. Both KF and LSE+VR reproduce the delay structure well, without introducing an appreciable phase shift. The slight asymmetry of the reconstructed curves with respect to zero lag is an expected feature of this cross-statistic: the estimators are causal, operating on current and past measurements only, so their output does not correlate symmetrically with the past and the future of the reference signal. The correlation peak remains located at τ=0, confirming that no appreciable artificial time lag is introduced. In terms of correlation amplitude, LSE+VR performs comparably to KF, following the reference more closely over part of the lag range. By contrast, cubic interpolation yields a markedly damped and distorted autocorrelation shape, indicating that the interpolation procedure alters the underlying dynamics.

In the channel-flow case, the autocorrelation decays more smoothly and monotonically, consistent with the broader-band character of the separated-flow dynamics. The differences between the reconstruction methods are smaller than in the jet case. LSE+VR provides the best agreement in terms of correlation amplitude over much of the lag range, while cubic interpolation reproduces the overall correlation shape reasonably well. In this representation, the KF result does not exhibit a clear advantage over interpolation. While the Kalman filter enforces physical consistency, its reliance on a linear dynamical model leads to over-smoothing in the stochastic channel flow; here, local interpolation better captures the high-frequency fluctuations, despite lacking a physics-based formulation.

Overall, these results confirm that the data-driven estimators preserve the temporal organization of the reconstructed signal without introducing an evident artificial time lag, whereas cubic interpolation degrades the correlation shape and therefore distorts the underlying temporal dynamics.

### Global reconstruction error

Finally, a compact scalar measure of reconstruction accuracy is reported in Table 2. For the *n*-th snapshot and the *i*-th sampling point of the HR grid, the local reconstruction error is defined as

33 $$\begin{aligned} e_{i,n}=\left\| \hat{\textbf{u}}_n(\textbf{x}_i)-\textbf{u}^{\textrm{LOR}}_n(\textbf{x}_i)\right\| , \end{aligned}$$

where $\textbf{x}_i$ denotes the location of the *i*-th HR sampling point. Hatted quantities denote the estimated fields, while the superscript $\textrm{LOR}$ refers to the HR-LOR reference used throughout the paper. The overall error is then defined as the average normalized Root-Mean Squared Error (RMSE) of this local velocity error over the testing dataset:

34 $$\begin{aligned} \delta =\frac{1}{N_s\,U_{\textrm{ref}}}\sum _{n=1}^{N_s} \sqrt{\frac{1}{N_p^{\textrm{HR}}}\sum _{i=1}^{N_p^{\textrm{HR}}} e_{i,n}^2}, \end{aligned}$$

where $N_s$ is the number of testing snapshots and $U_{\textrm{ref}}$ denotes the reference velocity, namely $U_j$ for the jet and $U_{ch}$ for the channel-flow case.

The results reported in Table 2 show that, over the full resolved domain, all three proposed estimators outperform the cubic-interpolation baseline in both configurations. Among the proposed methods, LSE provides the lowest overall error in both the jet and channel-flow cases, with LSE+VR yielding nearly identical values. The improvement over cubic interpolation is appreciable, although still moderate in absolute terms. This should be interpreted in light of the intrinsic limitations of a pointwise scalar metric. By compressing all spatial and temporal information into a single number, δ cannot discriminate between physically consistent reconstructions and fields contaminated by non-physical artefacts. The metric δ should accordingly be regarded as a complementary summary indicator rather than the primary criterion for assessing reconstruction quality.

The apparent disparity between the field- and spectrum-based diagnostics, where KF often provides the closest agreement with the reference, and the global error, where LSE prevails, can be interpreted in relation to the nature of the estimators. The LSE operator is identified by minimizing the mean-squared error over the training data and is therefore near-optimal by construction with respect to δ-type metrics; this optimality is achieved at the cost of a systematic attenuation of the fluctuations. The KF, by contrast, trades pointwise accuracy for dynamic and spectral consistency, which is rewarded by the energy-based diagnostics but not by a pointwise scalar error. Additionally, to assess the sensitivity of δ to the choice of the spatial domain, the metric has also been evaluated restricting the spatial average to the most energetic regions of the flow, defined as the HR grid points where the reference TKE exceeds a fraction α of its maximum; the resulting values $\delta _{\alpha }$, together with the corresponding mask coverages, are reported in Table 2 for α=0.25 and α=0.5. The errors increase for all methods, as expected since the metric remains normalized by $U_{\textrm{ref}}$ while the local fluctuation intensity grows, and the LSE-based estimators remain the most accurate in the restricted domains as well, with LSE+VR marginally improving over standard LSE in the channel-flow case. The contrast between the model-based estimators and the interpolation baseline, however, narrows in the energetic regions and, for the strictest threshold in the jet case (α=0.5, isolating the thin high-TKE bands of the shear layers over 8% of the resolved points), the KF pointwise error becomes slightly larger than that of interpolation. This behaviour reflects the different objective of the estimators discussed above: the KF privileges dynamic and spectral consistency over pointwise optimality, retaining fluctuation amplitudes that a pointwise metric penalizes wherever energetic structures are slightly misplaced, whereas smoother estimates are favoured by δ even when they underrepresent the flow dynamics. The domain-restricted analysis thus provides quantitative confirmation of the complementary character of δ-type indicators noted above.

Finally, it is useful to quantify the error introduced by the low-order truncation alone to partially decouple the estimation error from the fidelity of the reference itself. Evaluated with the same metric of Eq. (34), but comparing the full HR fields with their HR-LOR projection over the testing dataset, the truncation error amounts to $\delta _{\textrm{LOR}} = 0.0550$ and 0.0540 for the jet and channel-flow case, respectively. These values represent lower bounds for the total deviation of any of the proposed estimators from the full HR fields and should be kept in mind when interpreting the errors of Table 2, which are computed with respect to the HR-LOR reference. More precisely, since the estimated fields lie in the retained subspace by construction, while the truncation residual is orthogonal to it, the deviation of the proposed estimators from the full HR fields is the root-sum-square of the two contributions, $\sqrt{\delta ^2+\delta _{\textrm{LOR}}^2}$, amounting for instance to ≈0.088 and ≈0.117 for LSE in the jet and channel-flow cases, respectively; this decomposition does not apply to the interpolation baseline, whose output is not confined to the retained subspace. Since the truncation rank is selected by the *elbow criterion* precisely so that the discarded modes are predominantly noise-dominated (Sect. 2.1), the HR-LOR reference acts as a denoised version of the HR fields, and $\delta _{\textrm{LOR}}$ partially reflects removed measurement noise; it should therefore be regarded as a conservative upper bound on the information excluded by the reference.

**Table 2:** Normalized RMSE δ (Eq. 34) for the two flow cases, computed over the full resolved domain and restricted to the most energetic regions, $\delta _{\alpha }$, defined as the HR grid points where the reference TKE exceeds a fraction α of its maximum (the spatial average of Eq. 34 being restricted to those points). The last row reports the fraction of resolved points retained by each mask. The best result in each column is highlighted in bold

	Jet flow			Channel flow		
Method	δ	$\delta _{0.25}$	$\delta _{0.50}$	δ	$\delta _{0.25}$	$\delta _{0.50}$
Cubic interpolation	0.0876	0.1235	0.1525	0.1193	0.1501	0.1672
KF	0.0792	0.1230	0.1632	0.1136	0.1460	0.1625
LSE	**0**.**0688**	**0**.**1073**	**0**.**1398**	**0**.**1042**	0.1345	0.1501
LSE+VR	0.0695	0.1075	0.1407	0.1043	**0**.**1338**	**0**.**1492**
Mask coverage	100%	32%	8%	100%	51%	29%

### Computational cost of the reduced-order refinement

To quantify the online overhead introduced by the proposed LR-to-HR refinement stage, a dedicated microbenchmark implemented in C++ was performed on the jet case. The benchmark was implemented following the online workflow of Method I (direct Kalman filter estimation), including the learned state-transition matrix $\textbf{F}$. Although the exact latent-state update depends on the chosen estimator, the benchmark is intended to provide a representative order-of-magnitude assessment of the additional computational overhead of the reduced-order refinement. The benchmark was carried out on a workstation running Microsoft Windows 11 Pro (build 26200), equipped with an AMD Ryzen Threadripper 7960X CPU (24 physical cores, 48 logical processors) and 256 GB of RAM. All reported timings correspond to single-threaded execution. The benchmark accounts only for the additional algebraic operations required once the LR velocity field is already available, namely: projection of the incoming LR field onto the reduced space, estimation of the HR latent state, and reconstruction of the HR field from the estimated latent coefficients.

**Fig. 13:**
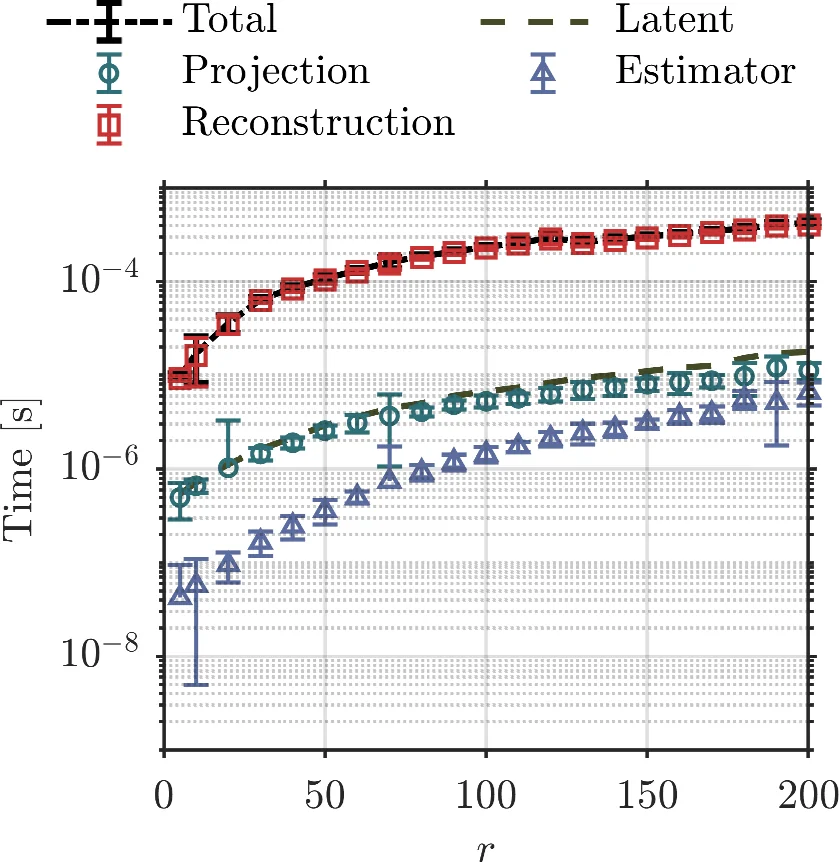
Computational cost of the additional online reduced-order refinement stage for the jet case as a function of retained rank r. The reported timings distinguish the contribution of the LR projection, latent-state estimation, and HR field reconstruction, together with their total cost. The line labelled *Latent* (i.e. operating only on the reduced-order POD coordinates) denotes the cost of operating only in the reduced space, i.e. projection plus latent-state estimation without full-field reconstruction. This quantity is particularly relevant for control-oriented implementations, in which the latent HR representation is sufficient and the full reconstructed field is not required online. Error bars denote the standard deviation over repeated runs

The results are reported in Fig. 13. For retained ranks up to r=200, the total added cost remains below 0.5 ms on average, with worst observed values below 0.8 ms. In the jet case, this overhead is comfortably below the 10 ms time budget associated with the 100 Hz update rate, indicating that the reduced-order refinement is not the limiting factor for real-time deployment. In the channel-flow case, the available time budget would be more restrictive because of the higher acquisition frequency. On the other hand, the number of spatial degrees of freedom $N_p^{\textrm{HR}}$ is lower by a few times, so the cost of the reduced-order operations is also expected to be correspondingly smaller.

The timing breakdown also clarifies the computational share of the steps of the process. The projection of the LR field and the latent-state estimation remain comparatively inexpensive, whereas the dominant contribution is the reconstruction of the full HR field. This trend is expected from the dimensionality of the three operations: the LR projection scales as $\mathcal {O}(r\,N_p^{\textrm{LR}})$, the latent-state estimation as $\mathcal {O}(r^2)$, and the full-field reconstruction as $\mathcal {O}(N_p^{\textrm{HR}}\,r)$. Since $N_p^{\textrm{HR}}\gg r$, the reconstruction step naturally dominates the total overhead.

This distinction is particularly relevant for control-oriented applications. When the objective is feedback estimation or low-order state monitoring, the online procedure can stop at the latent HR representation, without reconstructing the full field at each time step. In Fig. 13, this case corresponds to the line labelled *Latent*, which includes only the reduced space operations and excludes full-field reconstruction. Its cost remains of the order of $10\,\upmu \textrm{s}$, well below the characteristic time required for the LR velocity field estimation itself. The proposed framework is therefore especially attractive when the reduced coordinates, rather than the full reconstructed field, are the quantities of primary interest.

## Conclusions

A data-driven reduced-order framework has been proposed to estimate, in real time, high-resolution velocity fields and reduced-order flow coordinates from coarse real-time Event-Based Imaging Velocimetry (rt-EBIV) measurements. The approach was assessed on two broadband turbulent flow datasets, namely a submerged water jet and a channel flow over a square rib, using paired LR/HR data obtained from the same raw EBV recordings through two processing pipelines of different fidelity. As a result, the offline training stage does not require any modification of the experimental setup or additional instrumentation, since the information used to learn the estimator is extracted from the same measurement chain that is employed online.

Compared with direct upsampling with cubic interpolation of the LR measurements, the proposed estimators provide consistently more reliable reconstructions of fluctuation-related flow content. Their benefit is not limited to visually plausible super-resolution, but extends to a more physically consistent reconstruction of second-order statistics, temporal and spatial spectra, reduced-order modal dynamics, and temporal autocorrelation, while avoiding the grid-imprinting artefacts introduced by purely spatial upsampling. In this sense, the proposed framework should not be interpreted as a simple LR-to-HR interpolation operator, but rather as a reduced-order estimator that combines learned cross-resolution mappings with temporal regularization.

An important aspect of the methodology is that the reduced-order modelling stage also acts as a denoising mechanism. This is particularly relevant in the present context, where the online LR measurements are obtained from a deliberately lightweight single-pass processing chain, which is computationally efficient but more sensitive to noise than the offline multi-pass procedure used to construct the HR reference. The signature of this excess noise is directly visible in the spectral analyses of Sect. 4.4, where the raw LR spectra exhibit an elevated broadband floor at high frequencies and wavenumbers (Figs. 8 and 9). Direct interpolation propagates this noisy and under-resolved information to the finer grid, whereas the proposed estimators constrain the reconstruction to a learned reduced-order subspace. This effect is especially valuable when the reduced-order HR coordinates themselves are the quantities of interest, since operating directly in reduced space provides access to a compact and dynamically meaningful representation of the flow while filtering part of the measurement noise.

**Fig. 14:**
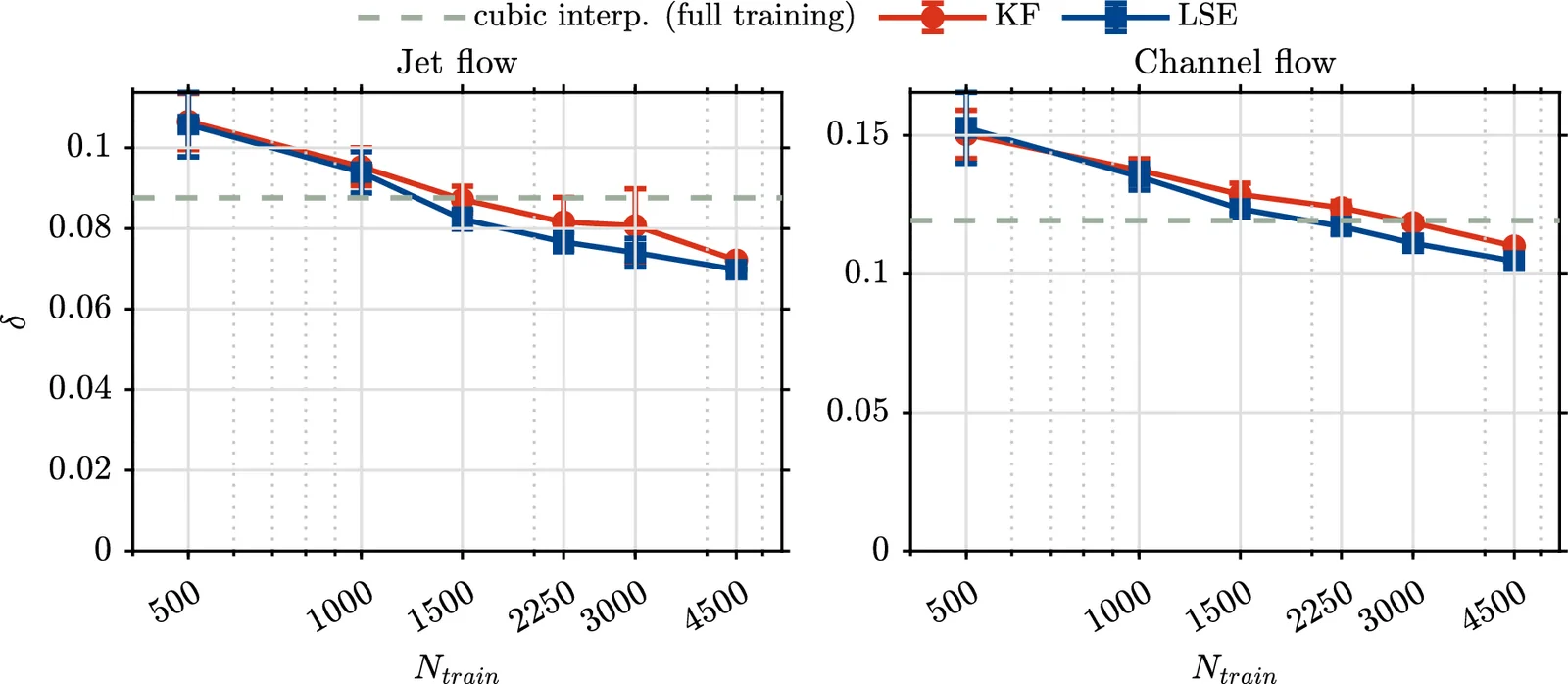
Global reconstruction error δ (Eq. 34, evaluated against the full-training HR-LOR reference) as a function of the training-dataset size, for the jet (left) and channel-flow (right) cases. Symbols and error bars denote the mean and standard deviation over the five training-window positions. The dashed line reports the full-training cubic-interpolation baseline of Table 2

Across the different diagnostics, no single estimator dominates uniformly in every representation. LSE yields the lowest global scalar error, LSE+VR remains very close to it and improves the recovery of fluctuation energy and higher-order content, while KF remains robust in several field- and spectrum-based comparisons. The variance rescaling correction should nevertheless be interpreted with care, since it is heuristic and identified from empirical statistics rather than derived from an optimal estimation principle. Its value is therefore mainly pragmatic: it compensates part of the systematic attenuation of standard LSE without changing the overall reduced-order estimation framework.

From a practical standpoint, the additional online cost of the refinement stage remains small compared with the real-time LR measurement itself. In particular, when only the reduced-order HR representation is required, the method can operate entirely in a reduced space at very low cost. The presence of a learned dynamical model also provides an additional practical advantage: if an LR measurement is temporarily unavailable, for example because of frame skipping, deliberate downsampling, or a transient processing bottleneck, the reduced HR state can still be propagated for a limited time using the model prediction alone. Although such a prediction-only estimate is less reliable than a measurement-corrected one, it remains preferable to a complete loss of the state estimate over short horizons.

The present methodology nevertheless remains tied to the operating conditions represented in the training data. In its current form, it should therefore not be regarded as a general solution for actuation-modified or strongly off-design flow regimes. This limitation should, however, be put in perspective. First, the offline training stage is required only once: its role is analogous to that of a calibration procedure, after which the measurement chain delivers offline-grade reduced-order estimates at real-time rates that the offline pipeline could not sustain. The cost of this calibration is quantified in Appendix A: training records of the order of $10^2$ convective time units suffice for the estimators to outperform the interpolation baseline, and about 300 convective time units recover the full performance reported in Table 2. This corresponds to less than one second of acquisition for the channel-flow case. The same train-offline/estimate-online paradigm underpins established real-time estimation frameworks such as SPPIV and its closed-loop applications (Kanda et al. 2022; Nonomura et al. 2025; Viguera et al. 2026). Second, the real-time monitoring and diagnostics of unactuated flows (e.g. continuous monitoring of experiments, detection of transients, or virtual sensing in reduced-order coordinates) constitutes a complete application domain in which the method is directly deployable in its present form. The one-off calibration is therefore performed only once before deployment: since tracking the reduced-order state requires storing only a few hundred coefficients per snapshot at negligible computational cost (Sect. 4.8), acquisitions substantially longer than the offline processing could afford become accessible, easing the convergence of statistics that require long records. Third, the framework is already applicable to a relevant class of flow-control problems, namely those in which the actuation does not significantly alter the dynamics within the sensed region. A notable example is the configuration in which sensing, actuation, and performance evaluation are located in distinct areas of the region of interest, so that the actuation affects the reward region but only marginally the sensed one, as in the real-time EBIV control experiment presented by Willert et al. (2026). Extending the framework towards actuation-aware estimation (i.e. towards fully closed-loop active-flow-control conditions in which the actuation modifies the sensed dynamics) and multi-regime training is a natural next step; a promising route, common in the flow-control literature, is to train the estimator over a basis of spanned actuation policies, so that the Kalman filter operates on a reduced-order representation valid across the actuated regimes. In addition, the present formulation relies on a linear reduced-order transition model and on a linear POD-based encoding. Future developments may therefore explore nonlinear extensions of both ingredients, for example through operator-inference strategies and nonlinear filtering frameworks such as the extended or unscented Kalman filter, which accommodate nonlinear state-transition and measurement models through local linearization and deterministic sampling of the state statistics, respectively, as well as nonlinear reduced-order representations based on autoencoders or related machine learning approaches. Finally, the development of a validated synthetic continuous- and pulsed-EBIV simulator (capable of reproducing the sensor behaviour under pulsed illumination of tracer particles, including per-pixel threshold dispersion, timing jitter, and arbiter saturation) would provide access to ground-truth-based assessments and represents, in the authors’ view, a valuable development for the event-based velocimetry community at large.

Within its current scope, however, the results demonstrate that deliberately coarse rt-EBIV processing can be used to extend the effective real-time operating range of the measurement chain towards higher sampling frequencies, while still recovering substantially richer and dynamically more consistent HR flow representations through reduced-order estimation. This is promising not only for high-quality real-time flow diagnostics, but also as a first step towards future observer-based and closed-loop flow-control applications.

## Data Availability

Data supporting this work are available on Zenodo at https://zenodo.org/uploads/20037404. The archive includes the .raw event-camera recordings, a subset of the corresponding pseudo-snapshots, and the processed velocity fields used in the present analysis. Additional data are available from the corresponding author upon reasonable request. A Python implementation of the real-time EBIV processing workflow and of the high-resolution estimation framework proposed in this work is available at https://github.com/LFranceschelli/VIBEstream. This repository is intended to document and reproduce the estimation methodology; it does not include the full C++ implementation used for the dedicated real-time benchmark.

## Appendix A: Sensitivity to the training-dataset size

The offline training stage of the proposed framework plays the role of a calibration procedure, and its practical cost is set by the length of the recording required to identify the reduced-order bases and operators. This appendix quantifies this cost by repeating the complete training procedure on progressively shorter portions of the training data.

For each training-set size $N_{train} \in \{500, 1000, 1500, 2250, 3000, 4500\}$, the KF and LSE estimators are trained on contiguous windows of snapshots. Five window positions are considered within the full-training range, except for the largest training-set size, for which only one window is available. For each window, the estimators are built using only the information available within that window, namely the mean flow, the POD bases, the truncation ranks selected with the *elbow criterion* described in Sect. 2.1, the mapping operators, and the noise covariances. The validation and testing subsets are kept fixed. Performance is evaluated using the metric of Eq. (34) against the same HR-LOR reference used throughout the paper, which is built from the full-training set. Therefore, $\delta (N_{train})$ quantifies how closely a shorter calibration reproduces the full-training product. The comparison is performed on the total velocity fields, so that errors in the mean-flow estimation from shorter records are also included.

The results are reported in Fig. 14. The error decreases monotonically with the training-set size, with a limited scatter across the window positions, and the two estimators behave very similarly. Both match the full-training cubic-interpolation baseline with training records of about 1500 snapshots for the jet and 2250–3000 for the channel flow: expressed in physical terms, records of the order of $10^2$ convective time units in both cases (i.e. 15–$30\,\textrm{s}$ of acquisition for the jet and 0.3–$0.6\,\textrm{s}$ for the channel flow). Approximately 300 convective time units recover the full-training performance of Table 2, corresponding to $45\,\textrm{s}$ of acquisition for the jet and to less than one second ($0.9\,\textrm{s}$) for the channel flow. At the shortest records considered (≈33 convective time units), the estimation degrades gracefully, with δ exceeding the interpolation baseline by roughly 20–30%.

Two remarks are due for a fair reading of these curves. First, δ is the most severe possible metric for the short-training estimators, since it combines the pointwise character of the metric discussed in Sect. 4.7—which favours smoother estimates over dynamically consistent ones—with the representational mismatch between the coarser training-window basis and the fixed full-training reference basis; the values at small $N_{train}$ should therefore be regarded as conservative bounds on the practical usability. Second, the truncation rank selected by the *elbow criterion* can oscillate significantly between training windows, with only a marginal impact on δ, indicating that the performance of the framework is robust with respect to the exact truncation choice. Overall, these results support the interpretation of the offline training stage as a lightweight, one-off calibration: a recording of the order of $10^2$ convective time units suffices to deploy estimators that outperform direct interpolation, with performance saturating towards the full-training level as the calibration record grows.

## Footnotes

1 Throughout this work, the terms *online* and *real time* are used interchangeably to denote processing performed concurrently with the experiment, at low latency. We note that, strictly speaking, real-time operation implies meeting a hard deadline dictated by the application (Stankovic 2002), whereas online operation only implies immediate, non-batch processing; the distinction is application-dependent and the relevant latency requirement here is set by the flow timescales to be resolved.

## Declarations

### AI and AI-assisted technologies in the writing process

During the preparation of this work, the authors used ChatGPT and Grammarly to improve the readability and language of this manuscript. After using this tool/service, the authors reviewed and edited the content as needed and took full responsibility for the publication’s content.
